# EFHD1 promotes osteosarcoma proliferation and drug resistance by inhibiting the opening of the mitochondrial membrane permeability transition pore (mPTP) by binding to ANT3

**DOI:** 10.1007/s00018-024-05254-8

**Published:** 2024-05-25

**Authors:** Xin Shen, Mengjun Ma, Rujia Mi, Jiahao Zhuang, Yihui Song, Wen Yang, Hongyu Li, Yixuan Lu, Biao Yang, Yinliang Liu, Yanfeng Wu, Huiyong Shen

**Affiliations:** 1 Department of Orthopedics, The Eighth Affiliated Hospital of Sun Yat-sen University, No. 3025 Shennan Zhong Road, Shenzhen, 518033 Guangdong China; 2 Center for Biotherapy, The Eighth Affiliated Hospital of Sun Yat-sen University, No. 3025 Shennan Zhong Road, Shenzhen, 518033 Guangdong China

**Keywords:** Osteosarcoma, Chemoresistance, EFHD1, ANT3, mPTP

## Abstract

Chemoresistance is the main obstacle in the clinical treatment of osteosarcoma (OS). In this study, we investigated the role of EF-hand domain-containing protein 1 (EFHD1) in OS chemotherapy resistance. We found that the expression of EFHD1 was highly correlated with the clinical outcome after chemotherapy. We overexpressed EFHD1 in 143B cells and found that it increased their resistance to cell death after drug treatment. Conversely, knockdown of EFHD1 in 143BR cells (a cisplatin-less-sensitive OS cell line derived from 143B cells) increased their sensitivity to treatment. Mechanistically, EFHD1 bound to adenine nucleotide translocase-3 (ANT3) and inhibited its conformational change, thereby inhibiting the opening of the mitochondrial membrane permeability transition pore (mPTP). This effect could maintain mitochondrial function, thereby favoring OS cell survival. The ANT3 conformational inhibitor carboxyatractyloside (CATR), which can promote mPTP opening, enhanced the chemosensitivity of EFHD1-overexpressing cells when combined with cisplatin. The ANT3 conformational inhibitor bongkrekic acid (BKA), which can inhibit mPTP opening, restored the resistance of EFHD1 knockdown cells. In conclusion, our results suggest that EFHD1-ANT3-mPTP might be a promising target for OS therapy in the future.

**Supplementary Information:**

The online version contains supplementary material available at 10.1007/s00018-024-05254-8.

## Introduction

Osteosarcoma (OS) is the most common primary bone cancer in children, adolescents, and young adults [1]. While chemotherapy has been an essential strategy for treating OS since the 1970s, it has failed to improve overall survival rates in recent decades due to acquired and intrinsic resistance to drug therapy [2]. Hence, there is an urgent need to comprehend the mechanism underlying drug resistance in OS and to seek novel strategies for effective treatment.

The mitochondrial membrane permeability transition pore (mPTP) indicates a permeability change in the mitochondrial membrane, which was first observed in 1979 [3]. It has both physiological and pathological meanings for cells. Under normal conditions, mPTP selectively permits the free transport of molecules ≤ 1.5 KD. However, under pathological conditions, the mPTP loses selectivity and irreversibly opens, causing mitochondrial swelling, mitochondrial membrane potential (Δψm) decrease, and cytochrome c release, eventually promoting cell death [4, 5]. The natural structure of the mPTP is still unknown, but the widely accepted putative structure is assembled of voltage-dependent anion channels (VDACs) in the outer mitochondrial membrane (OMM), adenine nucleotide translocators (ANTs) in the inner mitochondrial membrane (IMM), and cyclophilin-D (CypD) in the matrix [6]. The mPTP has been described as a critical factor that is related to cell viability and death in many human diseases, such as cardiomyocyte ischemia/reperfusion injury [7], metabolic diseases, including insulin resistance and diabetes [8], and cancers, such as lung cancer [9] and breast cancer [10]. These findings confirmed that the mPTP is a promising drug target in human diseases and warrants further research.

EF-hand domain-containing protein 1 (EFhd1) is a mitochondrial inner membrane protein that contains two EF-hand motifs that can regulate the energy metabolism of mitochondria [11]. It was proven that it could manipulate neuronal and muscle cell apoptosis and differentiation [12]. In addition, a recent study confirmed that EFhd1 could regulate the morphology and energy metabolism of dorsal root ganglion neuron cell mitochondria [13]. This result suggested that EFhd1 plays an important role in mitochondrial metabolism. However, the specific mechanism is still unclear. Hou et al. have pinpointed that EFHD1 could activate mitoflashes by sensing the Ca^2+^ concentration of mitochondria [14]. As a transient, reversible increase in mitochondrial permeability, mitoflashes have been seen as a kind of transient opening of mPTP [15], and EFHD1 might work by regulating mPTP opening. However, there are few data showing that EFHD1 plays a role in tumor progression. Takane et al. suggested that the methylation levels of the EFHD1 promoter were decreased in the plasma of colorectal cancer patients [16]. This means that EFHD1 is worth in-depth study.

Adenine nucleotide translocase-3 (ANT3), which is one of the main structural proteins of the mPTP, is found in the IMM and functions in ADP/ATP transport in mitochondria [17]. ANT3 has two distinct forms: when ANT3 faces the mitochondrial matrix, it acquires the m-state and transports ATP to release ADP, and when ANT3 faces the cytoplasmic side, it acquires the c-state and transports ADP to release ATP; ANT3 achieves mitochondrial energy conversion through dynamic state transitions [18]. The different states of ANTs are consistent with the mPTP opening state, and mPTP opening requires ANTs to be placed in the c-state; however, when ANTs are fixed in the m-state, mPTP opening is inhibited [19]. The natural inhibitor carboxyatractylate (CATR) can lock ANT3 in the c-state, while bongkrekic acid (BKA) can place it in the m-state, thus facilitating or decreasing mPTP opening [20]. This suggests that the conformational changes of ANT3 play a crucial role in both mitochondrial metabolism and cell fate [21].

In our study, we found that EFHD1 could promote mitochondrial energy metabolism and chemoresistance in OS cells. Our results suggested that EFHD1 binds directly with ANT3, blocking mPTP opening and boosting mitochondrial function. As a result, EFHD1 promotes the survival of OS cells.

## Materials and methods

### OS sample collection

Thirty-two OS samples were collected from OS patients who underwent chemotherapy (AP, 25 mg/m^2^ doxorubicin, 100 mg/m^2^ cisplatin) at the Eighth Affiliated Hospital, Sun Yat-sen University, Guangdong, China. These tissues were frozen and preserved in liquid nitrogen. This study was approved by the Ethics Committee of The Eighth Affiliated Hospital, Sun Yat-sen University.

### Cell culture and establishment of cisplatin-less sensitive cell line

We obtained OS cell lines 143B, U2OS, MG63, Saos-2, and HEK293T embryonic kidney cell lines from the American Type Culture Collection (ATCC) and cultured them according to the protocol from ATCC. The cisplatin-less sensitive cell line was induced by treating the parental 143B cell line with cisplatin in a stepwise manner (from 0.01 to 0.5 µg/ml). After cells grew in medium with 0.5 µg/ml cisplatin, they were designated as cisplatin-less sensitive cell line and termed 143BR.

### Lentiviral infection

To construct stable OS cell lines, we cotransfected pLKO.1-shEFHD1, pGC-FU-EFHD1, hEFHD1-Del(1-89aa)-3FLAG-OE, hEFHD1-Del(90-125aa)-3FLAG-OE, hEFHD1-Del(126-161aa)-3FLAG-OE, and hEFHD1-Del(162-239aa)-3FLAG-OE plasmids into 293 T cells with psPAX2 and pMD2. G plasmids for 48 h. Initially, we seeded 293 T cells in a 75 cm^2^ cell culture dish and allowed them to reach 90% confluence. For Tube A preparation, we mixed 55 μl of Lipofectamine 3000 with 2 ml of Opti medium and incubated it at 37 °C for 5 min. Simultaneously, in Tube B, we mixed 4 μg of PSPAX2, 2 μg of PMD2G, 6 μg of the target plasmid, and 48 μl of P3000 with 2 ml of Opti medium. The contents of Tube A and Tube B were then combined and incubated at 37 °C for 20 min.

Next, we removed half of the medium from the 293 T cells and added the DNA-liposome complex to the remaining medium. After 12 h, we replaced the medium with fresh medium for the 293 T cells. After an additional 24 h, we filtered and collected the supernatant fractions, which were then used to infect 143B and 143BR cells using polybrene. Specifically, 143B and 143BR cells were seeded in six-well plates and cultured overnight. Subsequently, 1 ml of the supernatant fractions, along with 1 μl of polybrene, was added to each well. After 12 h, the supernatant fractions were replaced with fresh medium and the cells were further cultured for 24 h. Finally, the stable OS cell lines were selected using medium containing 2.5 μg/ml puromycin.

### RNA extraction and quantitative real-time PCR (qRT‒PCR)

According to the manufacturer’s instructions for the RNA-Quick Purification Kit (ES Science, China), OS cells were lysed, and total RNA was collected for cDNA synthesis. qRT-PCR was carried out by using SYBR Premix Ex Taq II reagents (TaKaRa), and the results were calculated based on the 2^−ΔCt^ method. The primer sequences are listed in Table S1. Experiments were performed three times (N = 3).

### Western blotting (WB)

We separated the total protein from OS cells using 10% SDS-PAGE and then transferred it to PVDF membranes. Then, the cells were blocked in 5% BSA for 1 h at room temperature. Then, the membranes were cultured with diluted primary antibodies (1:1000) overnight at 4 °C. The primary antibodies were as follows: ACTB (CST; #4970), c-Myc (CST; #13987), C-caspase 3 (CST; #9664), C-caspase 9 (CST; #20750), DYKDDDDK Tag (CST; #14793), IgG (CST; #5946), EFHD1 (SAB; #38043), ANT3 (SAB; #40595), ANT2 (SAB; #45754), BAX (SAB; #48690), COX IV (SAB; #49275), VDAC1 (SAB; #23076), and cytochrome C (ABclonal; #A4912). The membranes were then washed three times with TBST before incubation with a secondary antibody (1:5000; CST; #7074 and #7076) at room temperature for 2 h. A Bio-Rad ECL detection system was used to examine the signals, and ImageJ (version 1.8.0) was used to analyze the data. Experiments were performed three times (N = 3).

### Coimmunoprecipitation and LC‐MS/MS

Cells were harvested and lysed. The total protein was precleared with 20 μl of Dynabeads protein A (Invitrogen; #10004D) for 1 h at 4 °C. The precleared protein liquid was then incubated with antibody (2 μg/sample) for 4 h at 4 °C. Next, the protein solution was incubated with 20 μl of Dynabeads protein A overnight at 4 °C. After three washes with cold IP buffer, the beads were boiled, and the protein samples were collected for Western blotting. For LC‒MS/MS detection, the beads were eluted and analyzed at The Medical Research Center of Sun Yat-Sen Memorial Hospital, Sun Yat-Sen University, Guangzhou, China.

### Cell cycle analysis

OS cells were collected and resuspended in 75% ethanol and then incubated at −20 °C for 1 h. Next, Intracellular Staining Perm Wash Buffer (Biolegend, San Diego, USA) was used to permeabilize the cells overnight at 4 °C. After 3 washes, the samples were stained with PI (BD PharmingenTM; #550825) for 15 min. Cell cycle analysis was performed by a FACSort Flow Cytometer. Experiments were performed three times (N = 3).

### Annexin V/PI staining

We seeded OS cells in six-well plates and cultured them overnight. Then, we treated these cells with drugs (0, 5, 10 µg/ml cisplatin; 1 µM BKA; 1 µM CATR) for 2 days. Next, we harvested the cells and incubated them in the dark for 15 min at room temperature with staining solution (100 μL binding buffer, 5 μL Annexin V-FITC and 10 μL PI). The apoptosis rate was analyzed by a FACSort Flow Cytometer. Experiments were performed three times (N = 3).

### Cell viability assay

A total of 1 × 10^4^ cells were seeded in each well of 96-well plates. After 12 h of culture, cells were incubated with a series of concentrations of chemotherapy drugs for 1 day. Then, the medium was removed, and the cells were incubated with reaction solution (10 CCK-8 reagent + 100 µL medium) for 2 h in a cell culture incubator. The absorbance (450 nm) was measured by a microplate reader (VarioskanLUX, Thermo Scientific, USA). Experiments were performed three times (N = 3).

### Cell counting Kit-8 (CCK-8) assay

OS cells were seeded into 96-well plates at a density of 2 × 10^3^ cells/well and incubated for 24 h at 37 °C in 5% CO_2_. After 0, 1, 2, 3, and 4 days of cultivation, 10 µL CCK-8 reagent was added to each well followed by culture for 2 h at 37 °C in 5% CO_2_. Absorbance at 450 nm was measured by a microplate reader (VarioskanLUX, Thermo Scientific, USA). Experiments were performed three times (N = 3).

### Mitochondrial isolation

A Mitochondria Isolation Kit (Beyotime; #C3601) was used to isolate the mitochondria. Briefly, 5 × 10^7^ cells were collected and incubated with 2.5 ml of mitochondrial isolation reagent at 4 °C for 15 min. The cells were homogenized with a glass homogenizer (30 times) and then centrifuged at 600 × g for 10 min at 4 °C. The supernatant was collected and centrifuged at 11,000 × g for 10 min at 4 °C, and the precipitate (mitochondrial fraction) was collected for WB or mitochondrial swelling assay analysis. For the collected cytosolic fraction, the supernatant of the previous step was centrifuged at 12,000 × g for 10 min at 4 °C, and the supernatant (cytosolic fraction) was collected for WB analysis.

### Mitochondrial swelling assays

As described by Li [22], 2 mg of mitochondria was added to KCl media. Then, CaCl_2_ (500 nmol/mg mitochondrial protein) aqueous solution was added to the media to trigger mPTP opening. The degree of mitochondrial swelling was detected by measuring the absorbance (540 nm) decrease every 3 s for 10 min on a microplate reader (VarioskanLUX, Thermo Scientific, USA). Experiments were performed three times (N = 3).

### Determination of mPTP opening

Ninety-six-well plates were seeded with OS cells at a density of 1 × 10^4^ cells per well and treated with PBS or cisplatin (2 µg/ml) for 1 day. Then, the cells were incubated with medium containing 2 µM calcein-AM (Sigma-Aldrich; #17783) for 30 min in a cell culture incubator. The fluorescence of the cytosolic dye was quenched by adding 1 mM CoCl_2_ (Macklin; #C804815) and incubating at 37 °C for another 30 min. Then, the images were photographed by the Leica DMi8 and analyzed by ImageJ (version 1.8.0). Experiments were performed three times (N = 3).

### TUNEL assay

As directed by the manufacturer’s protocol, apoptosis of OS cells was measured in paraffin-embedded OS samples using a One Step TUNEL Assay Kit (Beyotime; #C1086). The images were photographed by a Leica DMi8. Experiments were performed three times (N = 3).

### Mitochondrial stress analysis

XF96 cell culture microplates were seeded with OS cells at a density of 1 × 10^4^ cells per well. The XF96 probe plate was hydrated with 200 µl prewarmed XF hydration solution in a CO_2_-free incubator for 12 h. Next, the cell culture medium was replaced by XF medium and incubated in a CO_2_-free incubator for 1 h. Then, the OCR of OS cells was recorded by an XF96 cell energy metabolism analyzer with sequential treatment with 2 µM oligomycin, 2 µM FCCP, and 0.5 µM rotenone. Experiments were performed three times (N = 3).

### Extracellular acidification rate (ECAR) assays

The ECAR was detected using an XF96 Extracellular Flux Analyzer (Seahorse Bioscience). Briefly, OS cells (1 × 104 cells/well) were seeded into 96-well Seahorse XF96 culture plates in DMEM containing 10% FBS and were incubated at 37 °C in a non-CO_2_ incubator overnight. Next, ECAR measurements were performed according to the manufacturer’s instructions. Glucose, oligomycin and 2-deoxyglucose (2-DG) were added to the wells at the indicated time points at final concentrations of 10 mM, 10 μM and 50 mM, respectively, Experiments were performed three times (N = 3).

### Analysis of the ATP-ADP exchange rate

As previously described [23]. We seeded OS cells in 96-well plates at a density of 1 × 10^4^ cells per well. Then, the culture medium was replaced with buffer containing 2 mM ADP and 1 µM Magnesium Green™-AM (Invitrogen; #M3735). Fluorescence (505/535 nm) was recorded by a VarioskanLUX instrument (Thermo Scientific, USA). Experiments were performed three times (N = 3).

### ATP concentration measurement

An ATP luminescence 1-step assay kit (PerkinElmer; #6016731) was used to measure the ATP concentration. Briefly, 96-well plates with 1 × 10^4^ cells per well were cultured in a cell culture incubator overnight. Then, the culture medium was replaced with reaction reagent. The luminescence was recorded by a VarioskanLUX instrument (Thermo Scientific, USA). Experiments were performed three times (N = 3).

### Transmission electron microscope (TEM)

We fixed OS cells with 2.5% glutaraldehyde at 4 °C for 4 h and then sent the samples to Servicebio (Wuhan, China) for further processing and analysis. Experiments were performed three times (N = 3).

### Confocal microscopy

We transfected the pDsRED2-Mito plasmid into OS cells and seeded them in confocal dishes. After treatment with PBS or cisplatin, 4% paraformaldehyde was used to fix the cell samples. The images were photographed by Nicon C2. Mitochondrial morphology was analyzed by ImageJ MiNA [24]. Experiments were performed three times (N = 3).

### Mitochondrial membrane potential (Δψm) and ROS production

OS cells were treated with PBS or cisplatin for 1 day, and then 1 µM TMRE or 1 µM MitoSOX was added to stain the cells at 37 °C for 30 min. Next, the cells were harvested to measure the Δψm and ROS production by FACScan flow cytometry. Experiments were performed three times (N = 3).

### Mitochondrial Ca2^+^ measurement

Mitochondrial Ca2^+^ levels were measured by Rhod-2 AM staining following the manufacturer’s protocols. Luminescence was recorded by a VarioskanLUX instrument (Thermo Scientific, USA). Experiments were performed three times (N = 3).

### Proliferation detection

According to the manufacturer’s instructions, 10 µM EdU staining reagent (Beyotime, #C0078S) was added to the medium for 2 h. Then, the cells were fixed and photographed by a Leica DMi8. Experiments were performed three times (N = 3).

### Xenograft tumor model

All animal experiments were approved by the Sun Yat-sen University Laboratory Animal Care and Use Committee (Guangzhou, China). Six-week-old athymic nude mice (n = 5/group) were used to establish a xenograft tumor model by subcutaneous injection of 1 × 10^7^/100 µl OS cells into the dorsi of mice. After 1 week of injection, we used calipers to measure the tumor volume every 3 days. Drug treatment (cisplatin, 3 mg/kg/2 days; CATR, 1 mg/kg/day; BKA, 1 mg/kg/day) was started in the second week by i.p. injection. The tumor volume (mm3) = ab^2^/2. To collect tumors, all mice were euthanized at 30 days.

### Immunohistochemistry (IHC)

IHC staining was performed by following the protocol of the SP Rabbit 307 & Mouse HRP Kit (ComWin Biotech, China). The percentage of positive cells was scored as follows: 0, no positive cells; 1, ≤ 10% positive cells; 2, 10–50% positive cells; and 3, > 50% positive cells. Staining intensity was scored as follows: 0, no staining; 1, weak staining; 2, moderate staining; and 3, dark staining. The comprehensive score (0, 1, 2, 3, 4, 6, 9) = staining percentage × intensity.

### Statistical analysis

All experiments were performed at least three times, and the results were analyzed by GraphPad PRISM 8. All data are presented as the means ± standard deviations (SD). Differences between groups were estimated by the t test or one-way ANOVA, with P < 0.05 indicating statistical significance.

## Results

### EFHD1 expression levels in OS are positively related to chemotherapy resistance

**Fig. 1:**
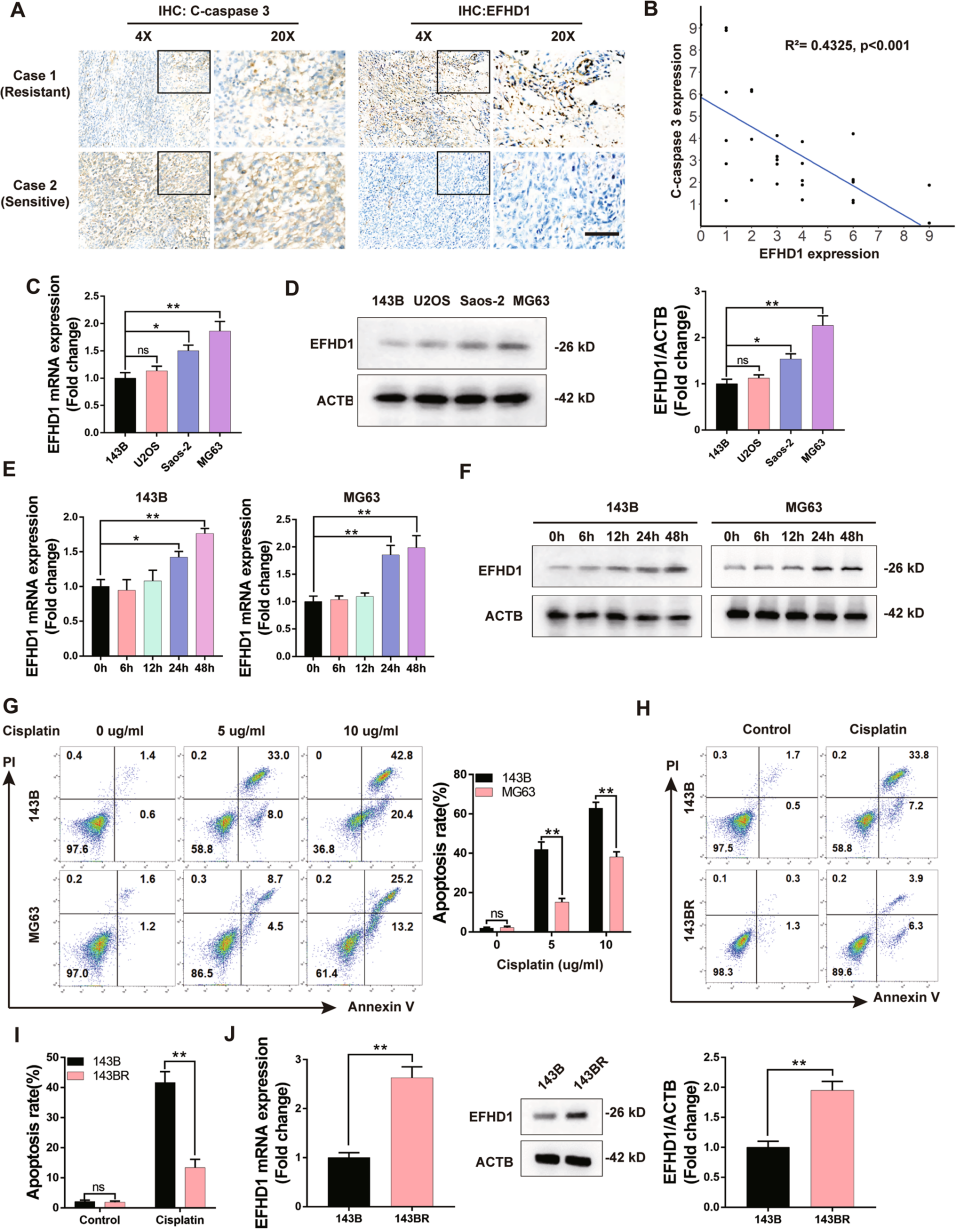
The expression level of EDHD1 is associated with the effectiveness of chemotherapy on OS. **A** Representative IHC staining photographs of OS samples. Scale bar = 20 µm, N = 32. **B** Scatterplot showing the negative correlation between EFHD1 and C-caspase 3 expression, N = 32. **C** qPCR examination of the mRNA levels of EFHD1 in OS cell lines, N = 3. **D** WB examination of the protein levels of EFHD1 in OS cell lines, N = 3. **E** and **F** mRNA levels (**E**) and protein levels (**F**) of EFHD1 in 143B and MG63 cells after treatment with cisplatin, N = 3. **G** Flow cytometric analysis of apoptosis in 143B and MG63 cells after cisplatin treatment for 2 days, N = 3. **H**, **I** Flow cytometric analysis of apoptosis in 143B and 143BR cells after cisplatin treatment for 2 days, N = 3. **J** EFHD1 mRNA and protein expression levels in 143B and cisplatin-resistant 143BR cells, N = 3. The data are presented as the means ± SEMss; *P < 0.05, **P < 0.01

Because the function of EFHD1 is still unclear, we investigated the possible role of EFHD1 in OS chemoresistance. We collected 32 specimens from OS patients who underwent chemotherapy (AP, 25 mg/m^2^ doxorubicin, 100 mg/m^2^ cisplatin) and subjected these samples to IHC staining. As Fig. 1A, B shows, the levels of C-caspase 3 were inversely associated with EFHD1 expression. To investigate whether the EFHD1 expression levels were associated with chemoresistance in OS cell lines, we first assessed endogenous EFHD1 expression in four OS cell lines. We found that MG63 cells exhibited approximately twofold higher expression of EFHD1 than 143B cells, which had the lowest levels (Fig. 1C, D). Meanwhile, we isolated their mitochondria and assessed EFHD1 expression at the mitochondrial level. We observed that MG63 cells exhibited the highest EFHD1 expression, while 143B cells showed the lowest EFHD1 expression (Fig. S3A).Then, we treated 143B cells and MG63 cells with cisplatin and detected the change in EFHD1 expression at different time points. The results showed that the expression of EFHD1 could be strongly induced by cisplatin in 143B and MG63 cells (Figs. 1E, F, S1). Next, we treated 143B and MG63 cells with cisplatin (5, 10 µg/ml) for 48 h and assessed the apoptosis rate by Annexin V/PI staining. The data suggested that 143B cells showed a higher apoptosis rate than MG63 cells under treatment with both concentrations of cisplatin (Fig. 1G). Furthermore, we established a cisplatin-less-sensitive cell line, 143BR. The cell size and cell cycle were similar to those of the parental 143B cell line (Fig. S2A–D). However, the cell viability assay revealed that 143BR cells had a higher IC50 than 143B cells (Fig. S2E). These results were also confirmed by Annexin V/PI staining, as shown in Fig. 1H and I. Next, we compared the levels of EFHD1 between 143 and 143BR cells by qPCR and WB analysis. As Figs. 1J and S3B shows, 143BR cells expressed higher EFHD1 levels at both the mRNA and protein levels than 143B cells. These data indicate that the EFHD1 level in OS might be positively associated with resistance to chemotherapy.

### EFHD1 promotes OS proliferation and chemoresistance in vitro and in vivo

**Fig. 2:**
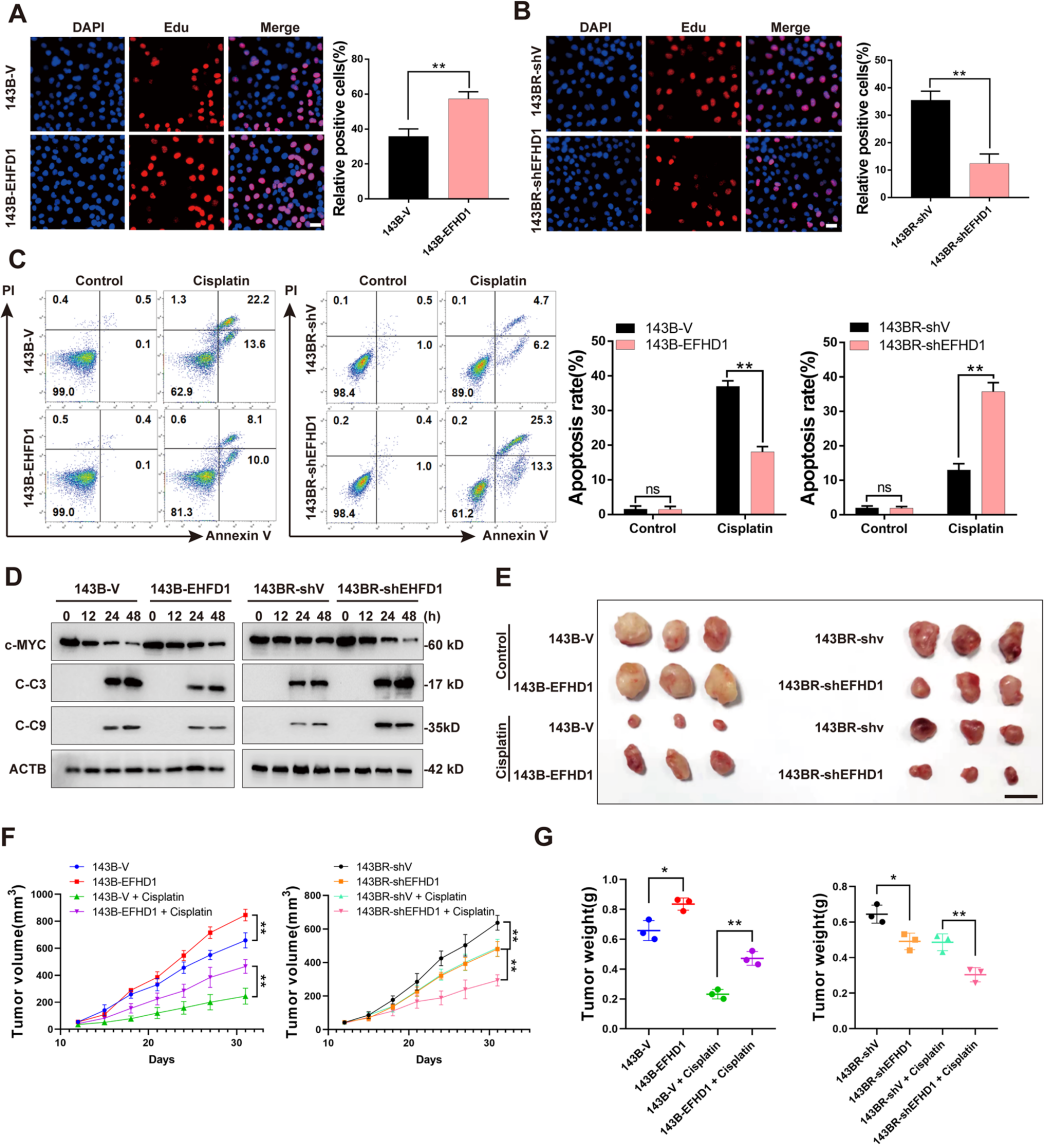
EFHD1 promotes OS proliferation and chemoresistance in vitro and in vivo. **A** The proliferation of 143B-V and 143B-EFHD1 cells was detected by EdU staining, N = 3. **B** The proliferation of 143BR-shV and 143BR-shEFHD1 cells was detected by EdU staining, N = 3. **C** Apoptosis of 143B-V, 143B-EFHD1, 143BR-shV and 143BR-shEFHD1 cells was measured by flow cytometric analysis, N = 3.** D** WB analysis of c-Myc, cleaved caspase 3 (C–C3) and cleaved caspase 9 (C–C9) expression in 143B-V, 143B-EFHD1, 143BR-shV and 143BR-shEFHD1 cells after cisplatin treatment, N = 3. **E**–**G** Tumor images (**E**), tumor volume (**F**) and tumor weight (**G**) of 143B-V and 143B-EFHD1 cell xenografts as well as 143BR-shV and 143BR-shEFHD1 cell xenografts with/without cisplatin treatment. Scale bar = 1 cm. N = 3 mice/group. The data are presented as the means ± SEMss; *P < 0.05, **P < 0.01

To explore the potential role of EFHD1 in OS cells, stable EFHD1-overexpressing and EFHD1-knockdown cell lines were established in 143B (143B-EFHD1) and 143BR (143BR-shEFHD1 and 143BR-EFHD1) cells, respectively, by lentiviral infection. Additionally, we constructed stable empty vector-carrying cell lines as controls in both 143B (143B-V) and 143BR (143BR-shV and 143BR-V) cells (Figs. S4A–C, S7A). Next, we measured their proliferation rate by EdU staining assay and Cell Counting Kit-8 (CCK-8) assay. The results showed that the 143B-EFHD1 cell line had more EdU-positive cells than the 143B-V cell line (Fig. 2A). Also, CCK8 results show that 143BR-EFHD1 cells exhibited higher proliferation rate than 143BR-V cells (Fig. S7B). In addition, the 143BR-shEFHD1 cell line had fewer EdU-positive cells than the 143BR-shV cell line (Fig. 2B). Then, we treated these cells with cisplatin and assessed the apoptosis rate by Annexin V/PI staining. We found that overexpression of EFHD1 in 143B and 143BR cells significantly reduced the apoptosis rate compared with that of the vector group. However, knockdown of EFHD1 dramatically elevated the apoptosis rate compared with 143BR-shV cells (Figs. 2C, S7C). Furthermore, we treated these cells with two other chemotherapeutic drugs, namely, adriamycin (ADR) and methotrexate (MTX), which are both commonly used for the treatment of OS in the clinic. As the cell viability assay data showed, 143B-EFHD1 cells exhibited a higher IC50 than 143B-V cells regardless of whether they were treated with ADR or MTX. 143BR-shEFHD1 cells exhibited a decreased IC50 compared with 143BR-shV cells after ADR and MTX treatment (Fig. S5A, B). Furthermore, we measured the expression of c-Myc, C-caspase 9, and C-caspase 3 at different time points. We found that the c-Myc levels in 143B-EFHD1 cells showed a smaller decrease than those in 143B-V cells. 143BR-shEFHD1 cells had a dramatic reduction in c-Myc expression compared with 143BR-shV cells. In addition, 143B-EFHD1 cells had lower levels of C-caspase 9 and C-caspase 3 than 143B-V cells. However, the expression levels in 143BR-shEFHD1 cells were greater than those in 143BR-shV cells (Figs. 2D and S6). To investigate the function of EFHD1 in vivo, we established a xenograft model. In the control group, the EFHD1-overexpressing 143B cells resulted in increased tumor volume and tumor weight compared to 143B-V cells. Knockdown of EFHD1 in 143BR cells reduced tumor volume and tumor weight compared with 143BR-shV cells. After cisplatin treatment, the average tumor weight of the 143B-EFHD1 group was decreased by approximately 44% (from 0.84 g to 0.47 g) compared with that of the control group. However, this figure of the 143B-V group was significantly reduced by approximately 65% (from 0.65 g to 0.23 g). On the other hand, the 143BR-shEFHD1 and 143BR-shV groups showed decreases of approximately 39% (from 0.49 g to 0.30 g) and 25% (from 0.64 g to 0.48 g), respectively (Fig. 2E–G). These results suggest that EFHD1 could promote OS cell proliferation and favor OS cell survival in vitro and in vivo.

### EFHD1 promoted mitochondrial functions

**Fig. 3:**
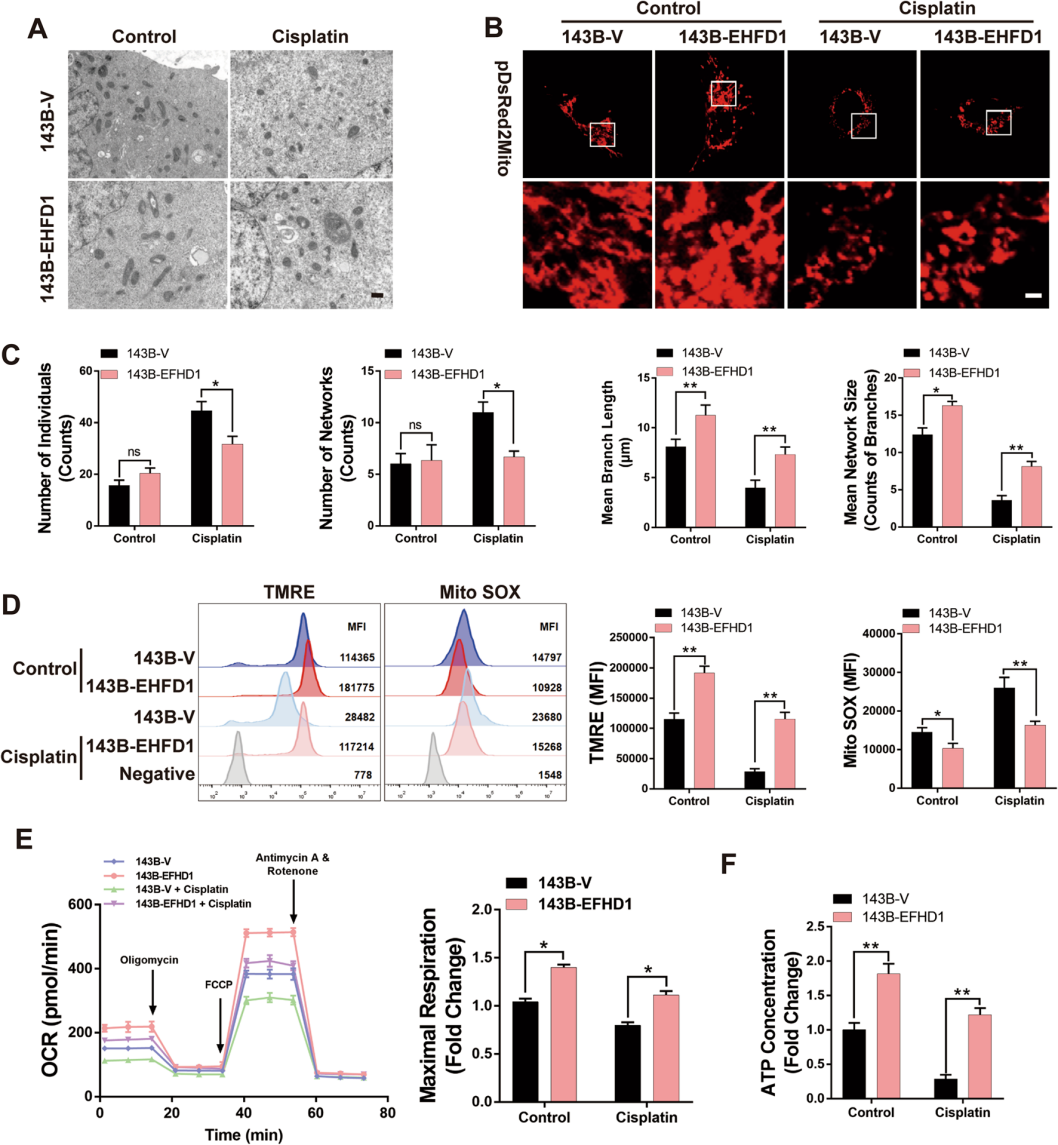
Overexpression of EFHD1 in 143B cells increases mitochondrial function. **A** TEM images showing the mitochondrial morphology of 143B-V and 143B-EFHD1 cells. Scale bar = 1 µm, N = 3. **B** CLSM images showing the mitochondrial morphology of 143B-V and 143B-EFHD1 cells. Scale bar = 1 µm, N = 3. **C** Histogram showing the results of mitochondrial network analysis of CLSM images, N = 3. **D** Mitochondrial membrane potential (TMRE staining) and ROS production (MitoSOX staining) of 143B-V and 143B-EFHD1 cells were analyzed by flow cytometry, N = 3. **E** Left: The OCR of 143B-V and 143B-EFHD1 cells was measured by an XF96 cell energy metabolism analyzer with oligomycin, FCCP and rotenone + antimycin sequential treatment. Right: The maximum respiration of 143B-V and 143B-EFHD1 cells was recorded after FCCP uncoupling, N = 3. **F** ATP concentrations in 143B-V and 143B-EFHD1 cells were measured by an ATP luminescence 1-step assay under control and cisplatin treatment conditions, N = 3. The data are presented as the means ± SEMss; *P < 0.05, **P < 0.01

The previous section showed that EFHD1 could promote OS cell proliferation and drug resistance. However, the specific mechanism is still unknown. According to the localization of EFHD1 in the IMM, we hypothesized that it may mediate OS cell proliferation and chemoresistance by influencing mitochondrial functions. To test our hypothesis, we analyzed mitochondrial morphology by transmission electron microscopy (TEM) and confocal laser scanning microscopy. As Fig. 3A–C shows, 143B-EFHD1 cells mainly had larger, tubular, densely packed mitochondria than 143B-V cells in both the control and cisplatin groups. Furthermore, we obtained opposite results when comparing 143BR-shEFHD1 cells to 143BR-shV cells treated with or without cisplatin (Fig. S8A–C). Mitochondria are considered central to energy metabolism, and their morphology plays a crucial role in sustaining mitochondrial respiration and cell survival [25]. Hence, we examined the change in mitochondrial membrane potential (Δψm) and ROS levels by flow cytometric analysis. We found that 143B-EFHD1 cells had an increased Δψm and decreased ROS levels compared with 143B-V cells in the control and cisplatin groups (Fig. 3D). Compared with 143BR-shV cells, 143BR-shEFHD1 cells showed lower Δψm and higher ROS production (Fig. S8D). Next, we examined the metabolic output of 143B-EFHD1 and 143BR-shEFHD1 cells. We found that the maximum respiration was increased in 143B-EFHD1 cells compared to 143B-V cells in both the control and cisplatin groups (Fig. 3E). 143BR-shEFHD1 cells exhibited decreased maximum respiration compared with 143BR-shV cells in both groups (Fig. S8E). However, the ECAR results indicate no significant difference between 143B-V and 143B-EFHD1 cells, as well as between 143BR-shV and 143BR-shEFHD1 cells (Fig. S9). In addition, 143B-EFHD1 cells generated much more ATP than 143B-V cells in the control and cisplatin groups (Fig. 3F). However, knocking down EFHD1 in 143BR cells decreased the concentration of ATP in both groups (Fig. S8F). These results suggested that EFHD1 could promote mitochondrial functions to protect OS cells from chemotherapy stress.

### EFHD1 inhibited mPTP opening and reduced cytochrome c release from mitochondria

**Fig. 4:**
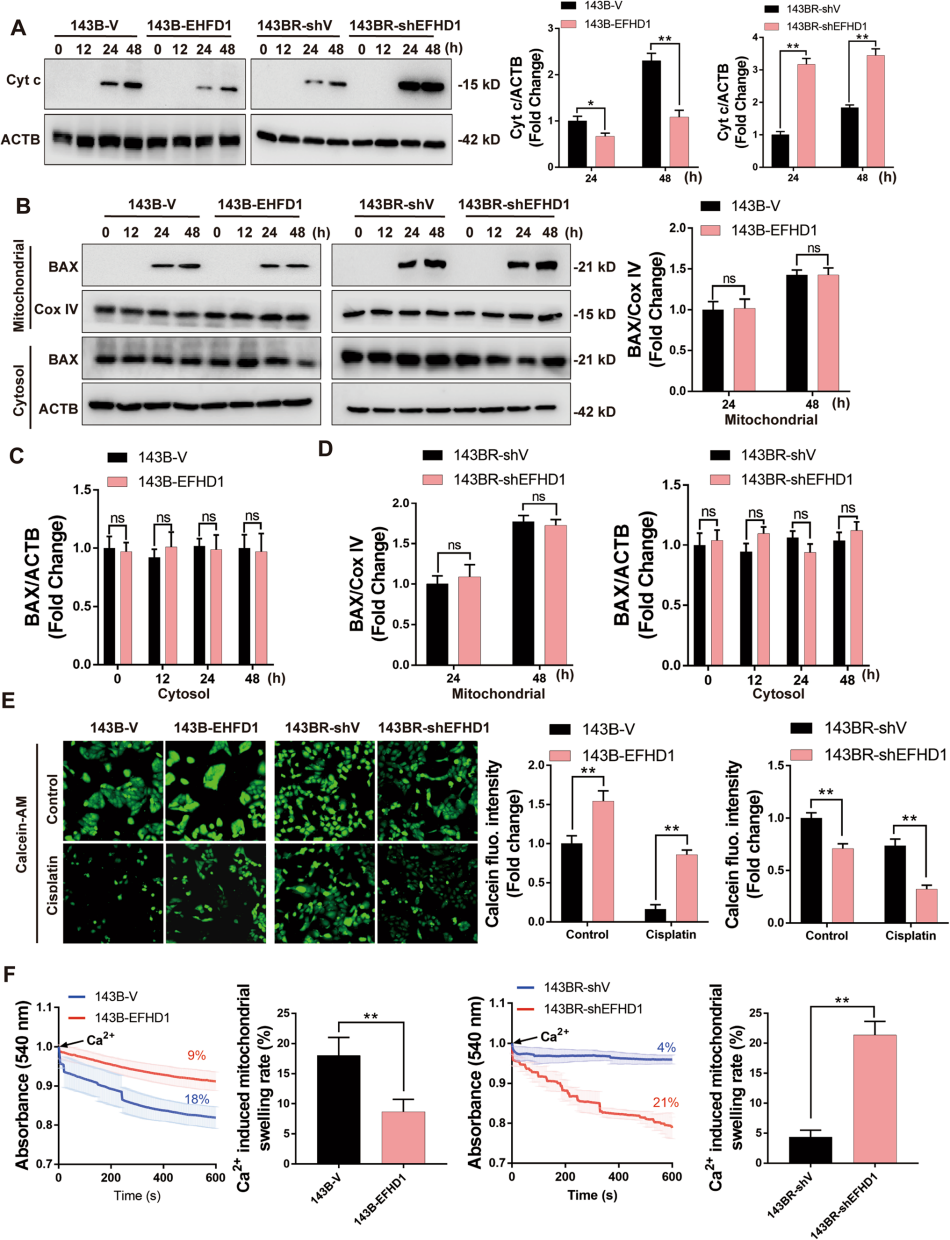
EFHD1 suppresses the opening of the mPTP and reduces the release of cyt c. **A** WB analysis of cytochrome c (Cyt c) release into the cytosol of 143B-V, 143B-EFHD1, 143BR-shV and 143BR-shEFHD1 cells induced by cisplatin, N = 3. **B**–**D** WB was used to detect BAX expression in the mitochondrial and cytosolic fractions of 143B-V, 143B-EFHD1, 143BR-shV and 143BR-shEFHD1 cells, N = 3. **E** The degree of mPTP opening in 143B-V, 143B-EFHD1, 143BR-shV and 143BR-shEFHD1 cells treated with/without cisplatin, N = 3. **F** Degree of Ca2^+^-mediated mitochondrial swelling in 143B-V, 143B-EFHD1, 143BR-shV and 143BR-shEFHD1 cells, N = 3. The data are presented as the means ± SEMss; *P < 0.05, **P < 0.01

Mitochondria are a central “hub” in regulating the intrinsic apoptotic pathway in human cells. Upon exposure to stimuli that induce apoptosis, such as chemotherapy, mitochondrial outer membrane permeabilization (MOMP) occurs and mPTP opening occurs, which are two key participants in mitochondrial apoptosis [26, 27]. To explore the mechanism by which EFHD1 contributes to chemoresistance, we first examined the levels of cytochrome c in the cytosolic fractions after treatment with cisplatin. As the data showed, 143B-EFHD1 cells significantly reduced the release of cytochrome c into the cytosol after cisplatin treatment. Moreover, 143BR-shEFHD1 cells released higher levels of cytochrome c than 143BR-shV cells (Fig. 4A). Next, we measured the degree of BAX translocation, which is a key step in MOMP. We found no significant difference between 143B-EFHD1 cells and 143B-V cells. Similar results were observed in 143BR-shEFHD1 and 143BR-shV cells (Fig. 4B–D). Therefore, we focused on other key participants in mitochondrial apoptosis. We assessed the opening of the mPTP in 143B-V, 143B-EFHD1, 143BR-shV and 143BR-shEFHD1 cells by the calcein-AM probe. We observed that EFHD1-overexpressing cells exhibited decreased mPTP opening compared with 143B-V cells and significantly decreased mPTP opening after treatment with cisplatin. Additionally, 143BR-shEFHD1 cells showed higher degrees of mPTP opening and dramatically increased mPTP opening after cisplatin treatment (Fig. 4E). According to Bonora et al., when facing uncontrolled stress, mPTP opens irreversibly, causing mitochondrial swelling and a decrease in Δψm [28]. Therefore, we detected the mitochondrial swelling rates and found that the mitochondrial swelling rates of 143B-EFHD1 (9%) and 143BR-shEFHD1 (21%) cells were noticeably lower or higher than those of 143B-V (18%) and 143BR-shV (4%) cells, respectively (Fig. 4F). These results suggested that EFHD1 could inhibit mPTP opening to support OS cell survival.

### EFHD1 inhibited mPTP opening by fixing ANT3 in the m-state

**Fig. 5:**
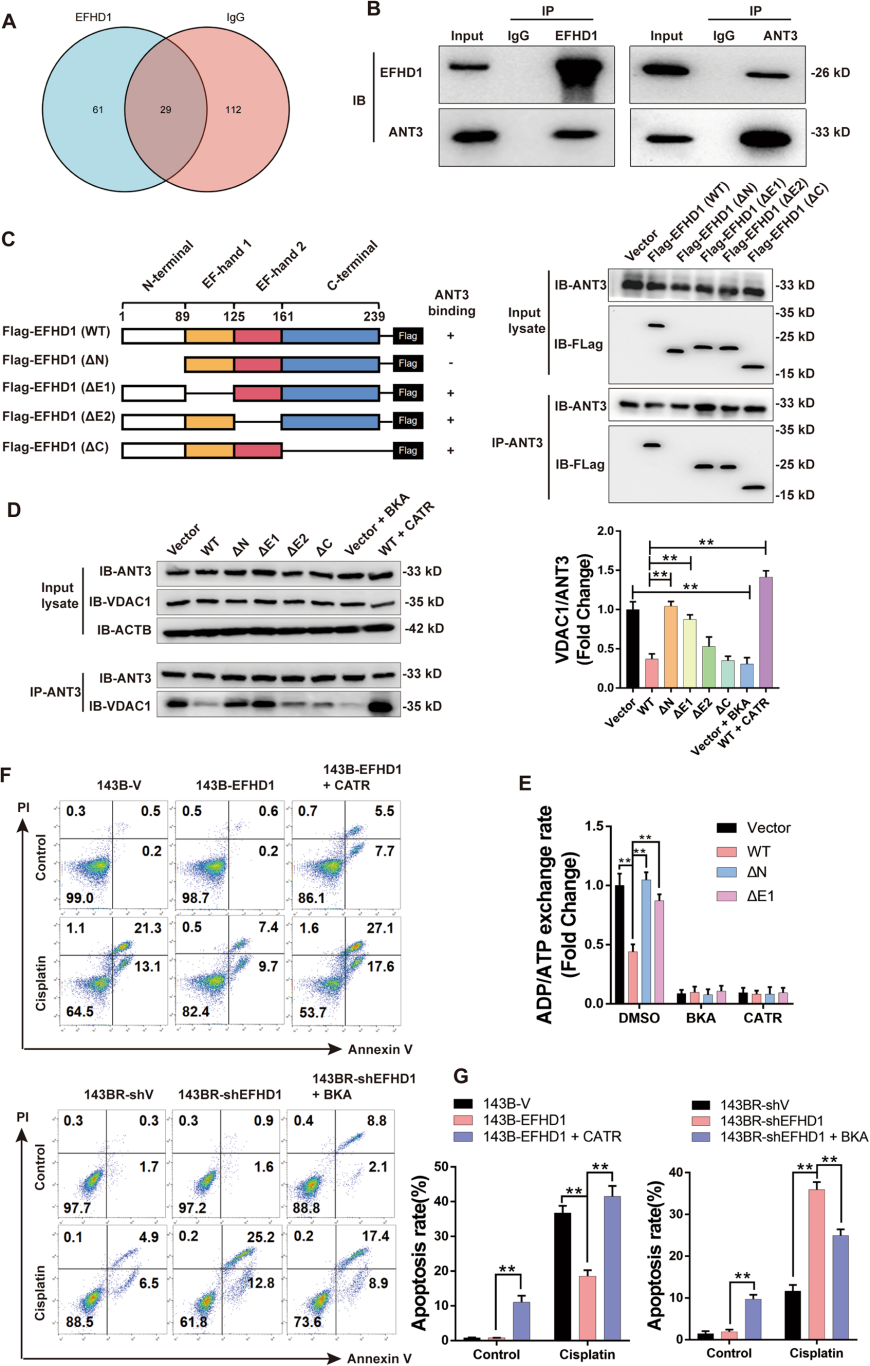
EFHD1 inhibits mPTP opening by inducing a conformational change in ANT3.** A** Venn diagram of the overlap of EFHD1 or IgG Co-IP binding proteins in 143B cells. **B** The binding of EFHD1 with ANT3 in 143B cells was analyzed by IP/IB, N = 3. **C** EFHD1 domain architecture and schematic representation of the respective deletion constructs used in this study that were expressed in 143B cells. The binding between Flag-tagged EFHD1 mutants and ANT3 was analyzed by IP/IB, N = 3. **D** IP/IB was used to detect the formation of the ANT3-VDAC1 complex in 143B cells, N = 3. **E** The ADP/ATP exchange rate of 143B cells was measured by Magnesium Green™, N = 3. **F** Left: apoptosis of 143B-V, 143B-EFHD1 and 143B-EFHD1 + CATR cells was measured by flow cytometric analysis; Right: apoptosis of 143BR-shV, 143BR-shEFHD1 and 143BR-shEFHD1 + BKA cells was measured by flow cytometric analysis, N = 3. **G** The apoptosis rate is shown in bar charts, N = 3. The data are presented as the means ± SEMss; *P < 0.05, **P < 0.01

To explore the specific mechanism of EFHD1 function, we first performed co-IP and liquid chromatography with tandem mass spectrometry (LC‒MS/MS) to identify the proteins that interact with EFHD1. We observed that EFHD1 could directly bind to ANT2 and ANT3, both of which are main structural components of the mPTP (Fig. 5A). We confirmed the results by Co-IP and WB analysis (Fig. 5B and S10). Then, we examined the changes in ANT2-VDAC1 and ANT3-VDAC1 complex formation in 143B-V and 143B-EFHD1 cells. Overexpression of EFHD1 attenuated the formation of the ANT3-VDAC1 complex but had no significant effect on ANT2-VDAC1 complex formation (Fig. S11A&B). These results suggested that EFHD1 may inhibit mPTP opening by mainly interacting with ANT3. To further explore the binding of EFHD1 and ANT3, we transfected the truncated form of Flag-EFHD1 plasmids into 143B cells. The results showed that only the truncated form of EFHD1 that retained its N-terminal region interacted with ANT3, suggesting that the N-terminal region is crucial for binding (Fig. 5C). The function of ANT3 is mainly mediated by conformational changes [21]. Therefore, we analyzed ANT3-VDAC1 complex formation, as shown in Fig. 5D. The data suggested that EFHD1 inhibited the formation of the ANT3-VDAC1 complex, similar to BKA. However, both the ΔN mutant and the ΔE1 mutant reversed this inhibitory effect. ANT3 is an ADP/ATP transporter. To further explore the effects of EFHD1 on ANT3 function, we detected the ADP/ATP exchange rate. As Fig. 5E shows, the ADP/ATP exchange rate of the WT group was significantly reduced compared with that of the vector group, while both the ΔN mutant and the ΔE1 mutant reversed this inhibitory effect. This suggested that EFHD1 could fix ANT3 in the m-state to prevent ANT3 from forming the mPTP complex.

The EF-hand motif is a Ca^2+^-binding motif. Ca^2+^ is one of the most important promoters of mPTP. Hence, we hypothesized that EFHD1 may induce a conformational change in ANT3 by directly binding to ANT3 via the N-terminal region and sensing changes in Ca2^+^ levels by EF-hand domain 1. According to Xu et al. [29], cisplatin could induce a significant Ca^2+^ influx into the mitochondrial matrix. We confirmed that cisplatin dramatically increased mitochondrial Ca^2+^ levels in OS cells (Fig. S12A). However, overexpressing EFHD1 did not alter mitochondrial Ca^2+^ concentrations in the control group, but an obvious decrease and increase were observed in the EFHD1-overexpressing group and EFHD1-knockdown group, respectively, compared with each vector group after cisplatin treatment (Fig. S12B, C). Thus, EFHD1 might play a protective role in Ca^2+^-induced cell death.

To explore whether ANT3 conformational changes were related to the EFHD1-mediated chemoresistance of osteosarcoma cells. We detected the apoptosis rate of 143B-EFHD1 and 143BR-shEFHD1 cells by treating them with the combination drugs. We found that CATR could improve the cisplatin sensitivity of 143B-EFHD1 cells, while BKA could increase the cisplatin resistance of 143BR-shEFHD1 cells. These results suggested that EFHD1 mediated OS chemoresistance by fixing ANT3 in the m-state to inhibit mPTP opening.

### Targeting the EFHD1-ANT3-mPTP axis enhances the efficacy of cisplatin in vivo

**Fig. 6:**
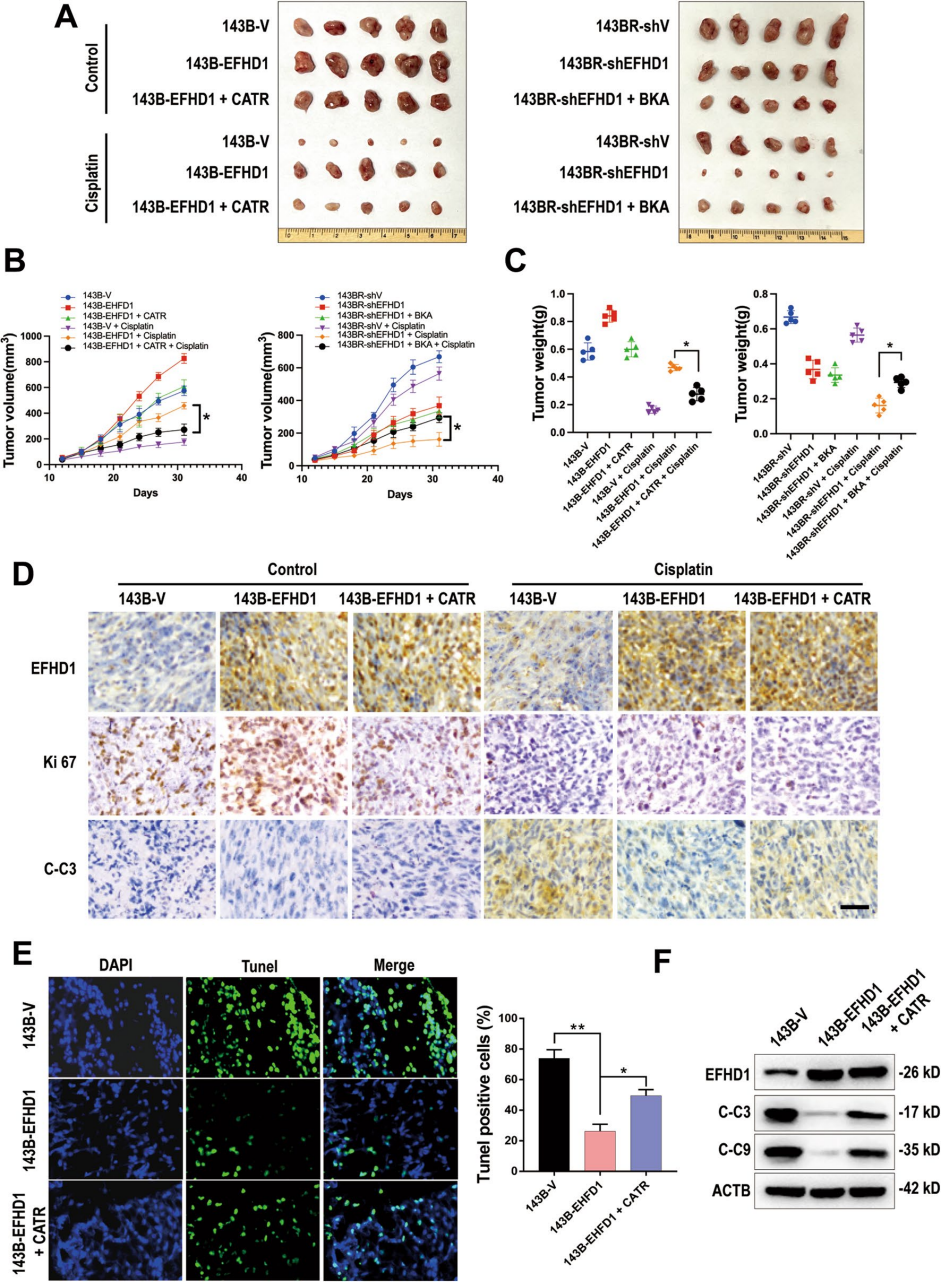
CATR enhances the efficacy of cisplatin in vivo.** A**–**D** Images of tumors (**A**), tumor volume (**B**) and tumor weight (**C**) of 143B-V and 143B-EFHD1 cell xenografts as well as 143BR-shV and 143BR-shEFHD1 cell xenografts that were treated as indicated. Scale bar = 1 cm. N = 5 mice/group. **D** IHC staining of Ki67 and C–C3 in 143B xenograft tumor samples. Scale bar = 20 μm, N = 5. **E** TUNEL assay was used to analyze apoptosis (scale bar = 50 μm), N = 5. **F** Apoptosis was analyzed by WB, N = 5. The data are presented as the means ± SEMss; *P < 0.05, **P < 0.01

To determine whether targeting the EFHD1-ANT3-mPTP axis can enhance the efficacy of chemotherapy drugs in vivo, we established a xenograft mouse model (Fig. 6A). Compared to the 143B-EFHD1-cisplatin group, we observed a considerable decrease in tumor volume (Fig. 6B) and tumor weight (Fig. 6C) in the 143B-EFHD1-cisplatin-CATR group. However, the combination of BKA and cisplatin in the 143BR-shEFHD1 group caused increased tumor volume (Fig. 6B) and tumor weight (Fig. 6C). Next, we performed IHC staining with EFHD1, Ki67 and C-C3 antibodies and found that CATR inhibited the proliferation of 143B-EFHD1 cells, while BKA promoted the proliferation of 143BR-shEFHD1 cells. Additionally, treatment with CATR combined with cisplatin increased C-C3 expression in the 143B-EFHD1 group compared to treatment with cisplatin alone. However, BKA decreased C-C3 expression in the 143BR-shEFHD1 + BKA + cisplatin group (Figs. 6D, S13, S15A&B). In addition, the apoptosis rate was significantly increased in the 143B-EFHD1-cisplatin-CATR group, as determined by a TUNEL detection kit. We also observed significantly enhanced levels of C-C3 and C-C9 in the 143B-EFHD1-cisplatin-CATR group compared to the 143B-EFHD1-cisplatin group (Figs. 6E&F, S14). Furthermore, BKA treatment decreased the apoptosis rate and C-C3 and C-C9 expression in the 143BR-shEFHD1-cisplatin-BKA group compared with the 143BR-shEFHD1- cisplatin group (Fig. S15C&D). Together, these results suggest that the EFHD1-ANT3-mPTP axis could be a promising target for OS therapy.

## Discussion

**Fig. 7:**
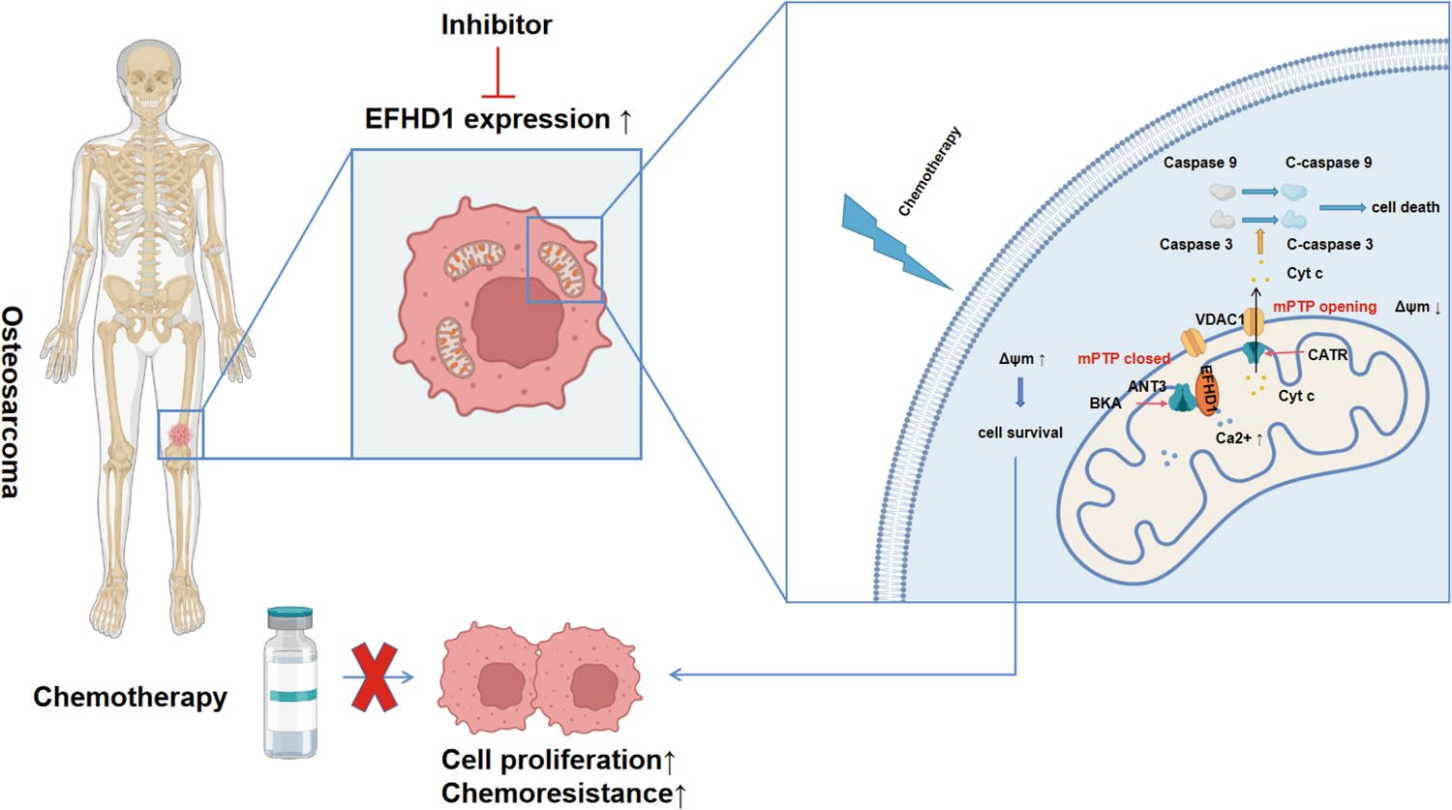
A schematic diagram showing that EFHD1 affects chemotherapy by regulating the opening of mPTP. EFHD1 expression is elevated in OS, causing OS cells to resist drug therapy. Mechanistically, EFHD1 maintains ANT3 in the m-state and prevents it from forming the mPTP complex, inhibiting the opening of the mPTP and favoring cell escape from death. CATR, a conformational inhibitor of ANT3, increases chemosensitivity in EFHD1-positive cells when used in combination with cisplatin. This result suggested that the EFHD1-ANT3-mPTP axis might be a promising target for OS therapy in the future

Chemoresistance is the main obstacle in cancer treatment, and most cancer treatments are hindered by their inability to induce cancer cell apoptosis due to adaptation responses [30]. When facing a hostile environment caused by drug therapy, cancer cells can quickly adapt and escape from death [31, 32]. The specific mechanism of stress adaptation responses is still largely unclear. Mitochondria, a kind of dynamic subcellular organelle, are a powerhouse of cell energy metabolism and a crucial regulator of cell death [25]. Many advanced studies have shown that mitochondria can quickly undergo dynamic changes, metabolism changes and cellular signaling pathway modulation, such as the intrinsic apoptotic pathway, to promote cancer cell survival in hostile environments [33]. Hence, targeting mitochondria to treat cancer and overcome chemoresistance has attracted increasing attention for various cancers. In our study, we found that EFHD1 can inhibit mPTP opening by fixing ANT3 in the m-state, promote mitochondrial functions and prevent cell death in a toxic environment, which contributes to promoting OS proliferation and drug resistance (Fig. 7).

EFHD1, which is a member of the EF-hand superfamily, has been less well studied in human cells. Some studies have shown that EFhd1 plays a crucial role in regulating cell growth and development processes. Including neuronal and muscle cell differentiation and programmed cell death [12]. Mitochondrial energy metabolism in dorsal root ganglion neurons [13]. This research suggests that EFHD1 might play a key role in mitochondrial metabolism and mitochondrial-mediated apoptosis. In our study, we noticed that EFHD1 expression was enhanced in OS tissue samples with chemoresistance, and increasing or decreasing EFHD1 levels in OS cells can inhibit or promote the toxicity of drugs in vivo and in vitro, respectively. Thus, targeting EFHD1 can be a promising therapeutic strategy for OS. Mitochondrial drug delivery systems are still immature and need further research.

mPTP is one of the most important participants in mitochondrial apoptosis, and it has been seen as a key factor in the development of many cancers [22]. Irreversible opening of mPTP leads to the release of many apoptosis promoters, such as AIF and cytochrome c, which activate caspases 3 and 9 by cleavage and ultimately cause cell death [34]. Previous studies have suggested that cancer cells can inhibit mPTP opening by decreasing the levels of mPTP inducers, such as Ca^2+^, lower ROS levels and induce glycolysis to adapt to toxic environments [35]. Therefore, targeting mPTP is considered a promising strategy for cancer therapy. However, identifying drugs that induce mPTP opening for use in clinical trials is very challenging due to the complex structural and regulatory mechanism of mPTP. In this study, we found that EFHD1 could inhibit mPTP opening, which contributes to promoting mitochondrial function and favoring cell survival. It provides another strategy to inhibit mPTP opening.

ANT3 belongs to the ANT family, which plays a crucial role in regulating metabolic reprogramming and cell death progression to promote cancer cell proliferation and drug resistance [17]. One study showed that ANT3 could significantly induce HeLa cell apoptosis and reduce tumor volume [36]. Notably, the conformational changes in ANT3 play a pathological role in determining the fate of tumor cells. CATR can fix ANTs in the c-state, which can induce mPTP opening, whereas BKA locks ANTs in the m-state, which inhibits the opening of mPTP [20]. This suggests that the c-state of ANTs is necessary for mPTP opening. Zhao et al. found that CATR can significantly increase the therapeutic effect of cisplatin in NPC cells by fixing ANT1 in the c-state [21]. We also obtained similar results in OS cells. However, no antitumor applications of CATR and BKA have been reported in the clinic, which might be due to their toxicity. Nevertheless, ANT3 is still a promising target for OS therapy, and further study is needed to find inhibitors.

In summary, this study suggests that EFHD1 plays a crucial role in OS cell proliferation and drug resistance by binding with ANT3 to inhibit the opening of mPTP. This confirms that EFHD1-ANT3-mPTP might be a promising target for overcoming the chemoresistance of OS cells in the future.

### Supplementary Information

Below is the link to the electronic supplementary material.

Supplementary file1 (DOCX 338 KB)

Supplementary file2 (XLSX 9 KB)

Supplementary file3 (XLSX 12 KB)

Fig S1 Quantification of EFHD1 expression based on WB. The data are presented as the means ± SEMs; *P <0.05, **P <0.01. Supplementary file4 (TIF 1229 KB)

Fig S2 143BR cells are resistant to cisplatin and do not exhibit changes in cell size or proliferation rate. A FSC values of 143B and 143BR cells were measured by flow cytometric analysis, N=3. B-D Cell cycle analysis of 143B and 143BR cells by PI staining and flow cytometric analysis, N=3. E Viability of 143B and 143BR cells after 24 h of cisplatin treatment, N=3. The data are presented as the means ± SEMs; *P <0.05, **P <0.01. Supplementary file5 (TIF 2864 KB)

Fig S3 The protein expression levels of EFHD1 in OS cell lines were examined using WB。A The protein expression levels of EFHD1 were examined using WB in the OS cell lines, N=3. B The protein expression levels of EFHD1 were examined using WB in 143B and 143BR, N=3. The data are presented as the means ± SEMs; *P <0.05, **P <0.01. Supplementary file6 (TIF 1365 KB)

Fig S4 qRT‒PCR and WB analysis of EFHD1 expression in overexpression and knockdown cells. A qRT‒PCR analysis of EFHD1 mRNA expression in 143B-EFHD1 and 143BR-shEFHD1 cells, N=3. B&C WB analysis of EFHD1 protein expression in 143B-EFHD1 and 143BR-shEFHD1 cells, N=3. The data are presented as the means ± SEMs; *P <0.05, **P <0.01. Supplementary file7 (TIF 2411 KB)

Fig S5 EFHD1 promotes osteosarcoma cell resistance to adriamycin and methotrexate treatment. A Viability of 143B-V and 143B-EFHD1 cells after 24 h of adriamycin treatment (left) and methotrexate treatment (right), N=3. B Viability of 143BR-shV and 143BR-shEFHD1 cells after 24 h of adriamycin treatment (left) and methotrexate treatment (right), N=3. The data are presented as the means ± SEMs; *P <0.05, **P <0.01. Supplementary file8 (TIF 2091 KB)

Fig S6 Quantification of c-Myc, cleaved caspase 9 (C-C9), and cleaved caspase 3 (C-C3) expression based on WB. The data are presented as the means ± SEMs; *P <0.05, **P <0.01. Supplementary file9 (TIF 1603 KB)

Fig S7 EFHD1 promotes 143BR proliferation and chemoresistance in vitro. A qRT‒PCR and WB analysis of EFHD1 expression in 143BR-V and 143BR-EFHD1 cells, N=3. B The proliferation of 143BR-V and 143BR-EFHD1 cells was detected by CCK8 proliferation assay, N=3. C Apoptosis of 143BR-V and 143BR-EFHD1 cells was measured by flow cytometric analysis, N=3. The data are presented as the means ± SEMs; *P <0.05, **P <0.01. Supplementary file10 (TIF 2109 KB)

Fig S8 EFHD1 knockdown in 143BR cells results in decreased mitochondrial function. A TEM images showing the mitochondrial morphology of 143BR-shV and 143BR-shEFHD1 cells treated with or without cisplatin. Scale bar=1 µm, N=3. B CLSM images showing the mitochondrial morphology of 143BR-shV and 143BR-shEFHD1 cells treated with or without cisplatin. Scale bar= 1 µm, N=3. C Histogram showing the results of mitochondrial network analysis of CLSM images. D Mitochondrial membrane potential (TMRE staining) and ROS production (MitoSOX staining) of 143BR-shV and 143BR-shEFHD1 cells treated with or without cisplatin were analyzed by flow cytometry, N=3. E Left: The OCR of 143BR-shV and 143BR-shEFHD1 cells was measured under basal conditions and in response to oligomycin, the mitochondrial decoupler FCCP and rotenone + antimycin after treatment with or without cisplatin. Right: the maximum OCR values were those achieved after FCCP uncoupling (maximum respiration), N=3. F ATP concentrations of 143BR-shV and 143BR-shEFHD1 cells were measured by an ATP lite 1step assay under control and cisplatin treatment conditions, N=3. The data are presented as the means ± SEMs; *P <0.05, **P <0.01. Supplementary file11 (TIF 6116 KB)

Fig S9 The ECAR of 143B-V, 143B-EFHD1, 143BR-shV and 143BR-shEFHD1 cells was detected using an XF96 Extracellular Flux Analyzer. The data are presented as the means ± SEMs; *P <0.05, **P <0.01, N=3. Supplementary file12 (TIF 1417 KB)

Fig S10 IP/IB analysis was used to detect the interaction of EFHD1 with endogenous ANT2 in 143B cells. The data are presented as the means ± SEMs; *P <0.05, **P <0.01, N=3. Supplementary file13 (TIF 2175 KB)

Fig S11 Overexpression of EFHD1 in 143B cells inhibits ANT3-VDAC1 complex formation. A IP/IB was used to detect the formation of the ANT3-VDAC1 complex in 143B-V and 143B-EFHD1 cells, N=3. B IP/IB was used to detect the formation of the ANT2-VDAC1 complex in 143B-V and 143B-EFHD1 cells, N=3. The data are presented as the means ± SEMs; *P <0.05, **P <0.01. Supplementary file14 (TIF 3782 KB)

Fig S12 EFHD1 reduces the cisplatin-induced accumulation of Ca2+ in the mitochondrial matrix. A The Rhod-2 AM probe was used to measure the mitochondrial Ca2+ concentrations in osteosarcoma cells after cisplatin treatment, N=3. B The Rhod-2 AM probe used to measure the mitochondrial Ca2+ concentrations in 143B-V and 143B-EFHD1 cells treated with/without cisplatin, N=3. C The Rhod-2 AM probe was used to measure the mitochondrial Ca2+ concentrations in 143BR-shV and 143BR-shEFHD1 cells treated with/without cisplatin, N=3. The data are presented as the means ± SEMs; *P <0.05, **P <0.01. Supplementary file15 (TIF 2174 KB)

Fig S13 Quantification of Ki-67 and C-C 3 expression based on IHC staining. The data are presented as the means ± SEMs; *P <0.05, **P <0.01, N=3. Supplementary file16 (TIF 1735 KB)

Fig S14 Quantification of Ki-67 and C-C 3 protein expression based on WB. The data are presented as the means ± SEMs; *P <0.05, **P <0.01, N=3. Supplementary file17 (TIF 1294 KB)

Fig S15 BKA decreases the sensitivity of 143BR-shEFHD1 cells to cisplatin in vivo. A&B IHC staining analysis of Ki-67 and C-C 3 expression in 143BR xenograft tissues. Scale bar, 20 μm, N=5. C Apoptosis was analyzed by TUNEL assay (scale bar, 50 μm), N=5. D Apoptosis was analyzed by Western blotting, N=3. The data are presented as the means ± SEMs; *P <0.05, **P <0.01. Supplementary file18 (TIF 9234 KB)

## Data Availability

The datasets supporting the conclusions of this article are available from the corresponding author on reasonable request.
