# Supplementary figures and images for: EFHD1 promotes osteosarcoma proliferation and drug resistance by inhibiting the opening of the mitochondrial membrane permeability transition pore (mPTP) by binding to ANT3
Source: Cell Mol Life Sci. 2024 May 25;81(1):236. doi: 10.1007/s00018-024-05254-8 (PMC11127909; doi:10.1007/s00018-024-05254-8)

**original acquired images from the PVDF membranes**

1. **Figure 4B, left**
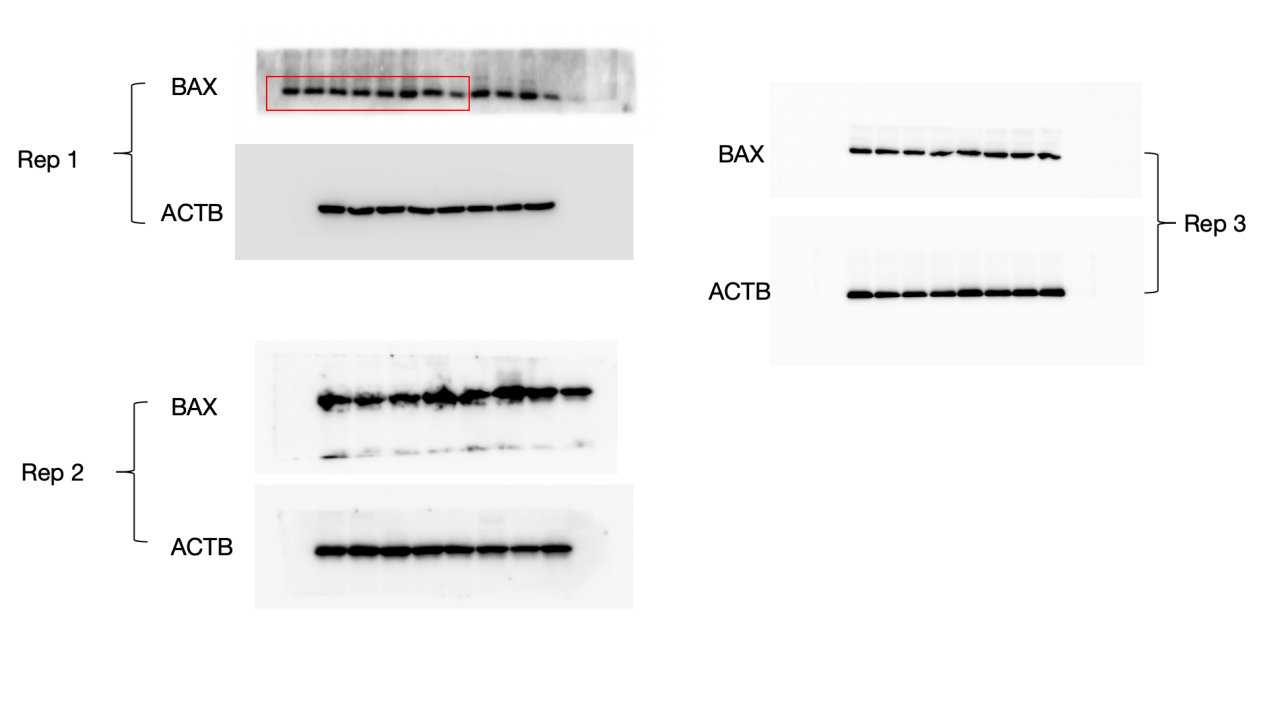

2. **Figure 4B, right**


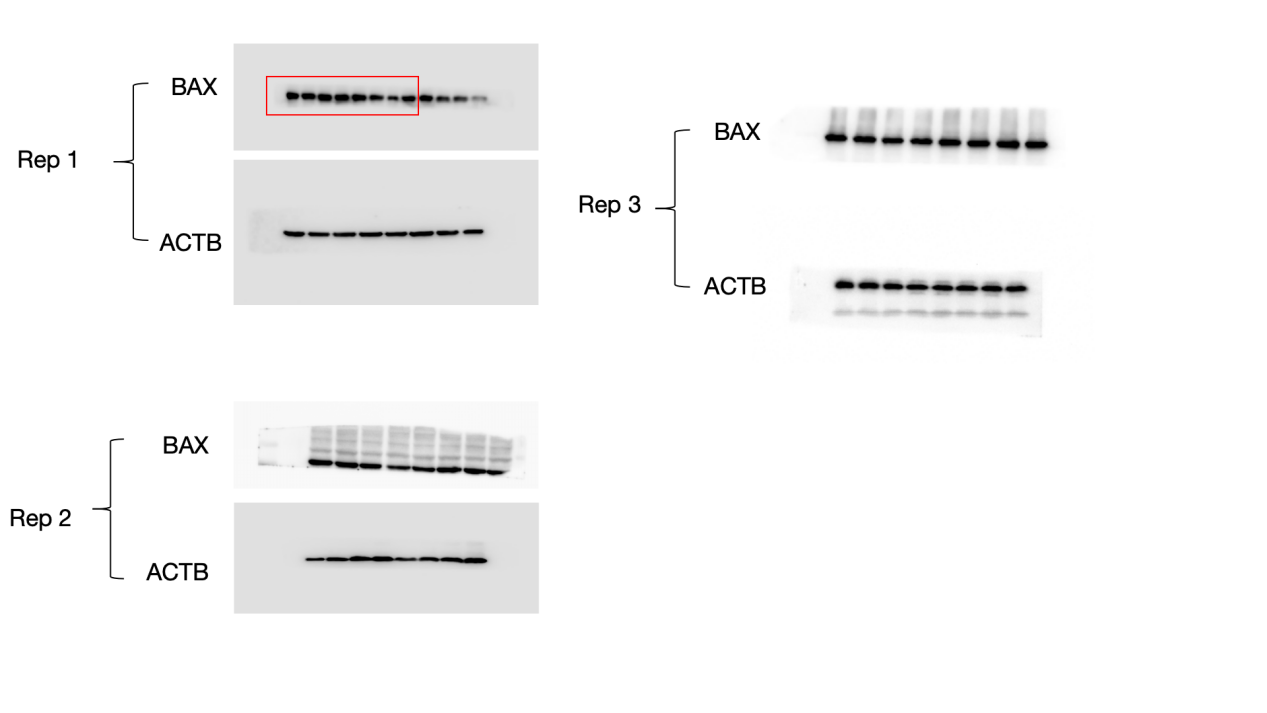

Supplement: Supplementary file 1 — Supplementary file1 (DOCX 338 KB) [file 18_2024_5254_MOESM1_ESM.docx]

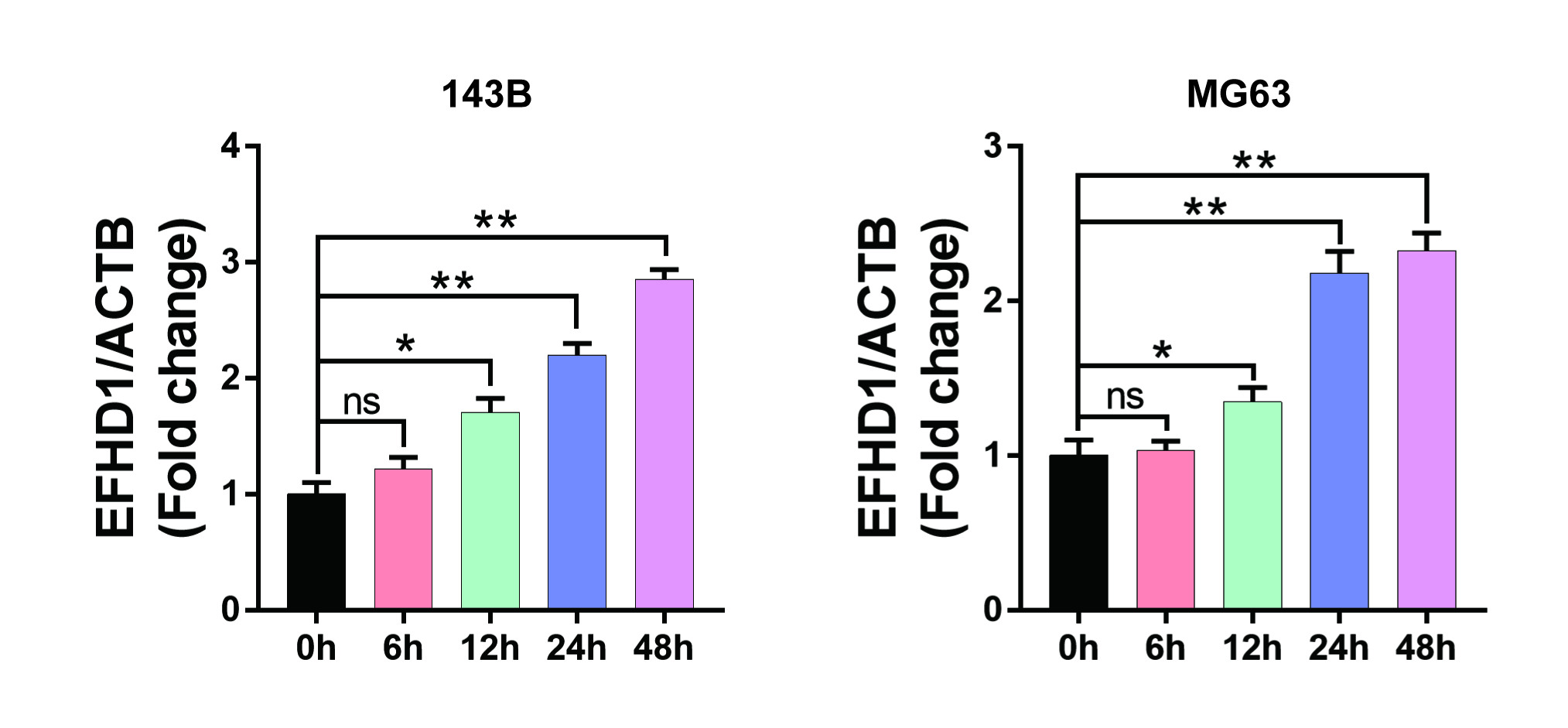

Supplement: Supplementary file 4 — Fig S1 Quantification of EFHD1 expression based on WB. The data are presented as the means ± SEMs; *P <0.05, **P <0.01. Supplementary file4 (TIF 1229 KB) [file 18_2024_5254_MOESM4_ESM.tif]

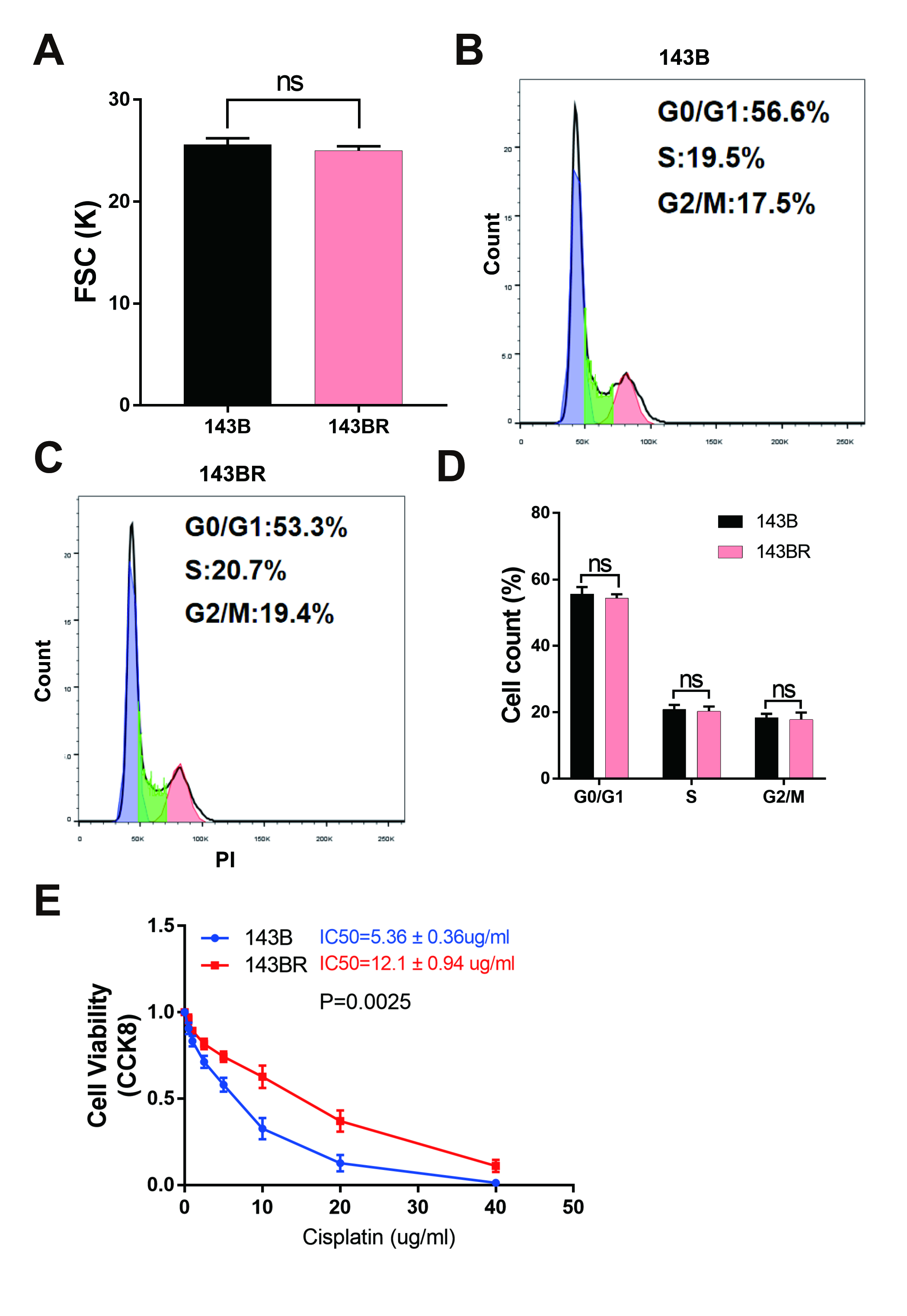

Supplement: Supplementary file 5 — Fig S2 143BR cells are resistant to cisplatin and do not exhibit changes in cell size or proliferation rate. A FSC values of 143B and 143BR cells were measured by flow cytometric analysis, N=3. B-D Cell cycle analysis of 143B and 143BR cells by PI staining and flow cytometric analysis, N=3. E Viability of 143B and 143BR cells after 24 h of cisplatin treatment, N=3. The data are presented as the means ± SEMs; *P <0.05, **P <0.01. Supplementary file5 (TIF 2864 KB) [file 18_2024_5254_MOESM5_ESM.tif]

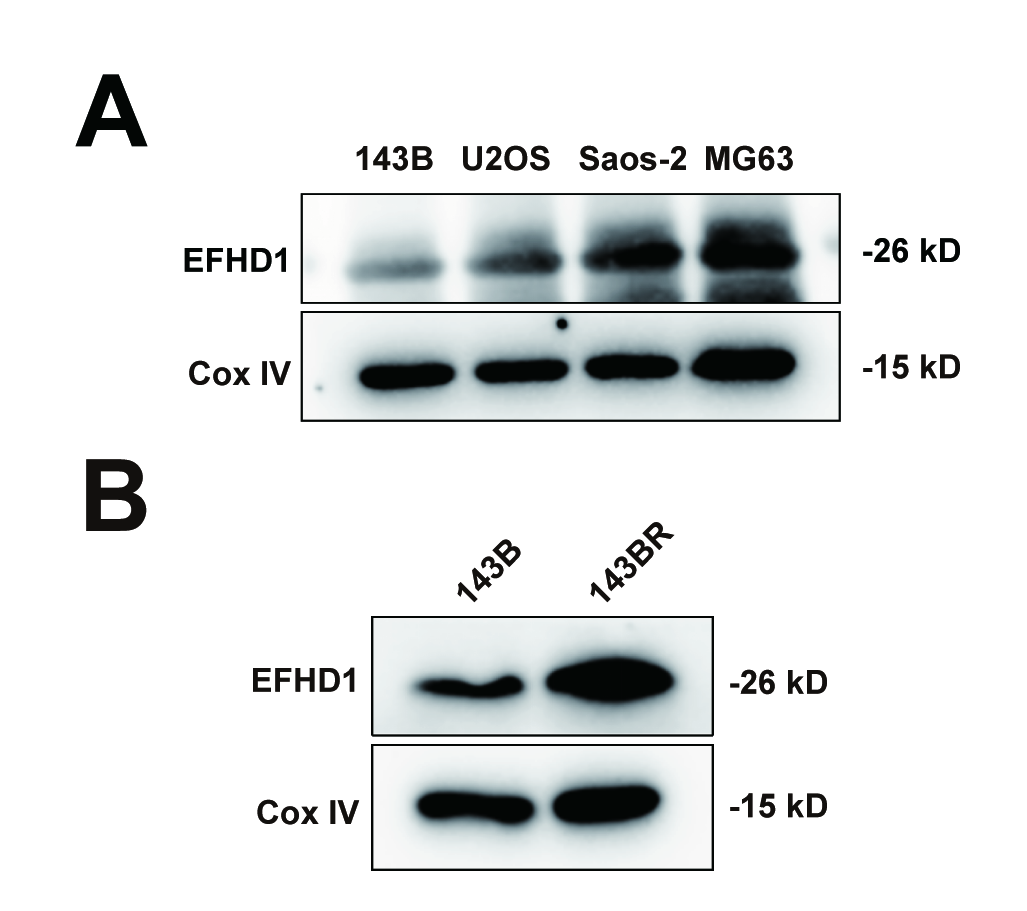

Supplement: Supplementary file 6 — Fig S3 The protein expression levels of EFHD1 in OS cell lines were examined using WB。A The protein expression levels of EFHD1 were examined using WB in the OS cell lines, N=3. B The protein expression levels of EFHD1 were examined using WB in 143B and 143BR, N=3. The data are presented as the means ± SEMs; *P <0.05, **P <0.01. Supplementary file6 (TIF 1365 KB) [file 18_2024_5254_MOESM6_ESM.tif]

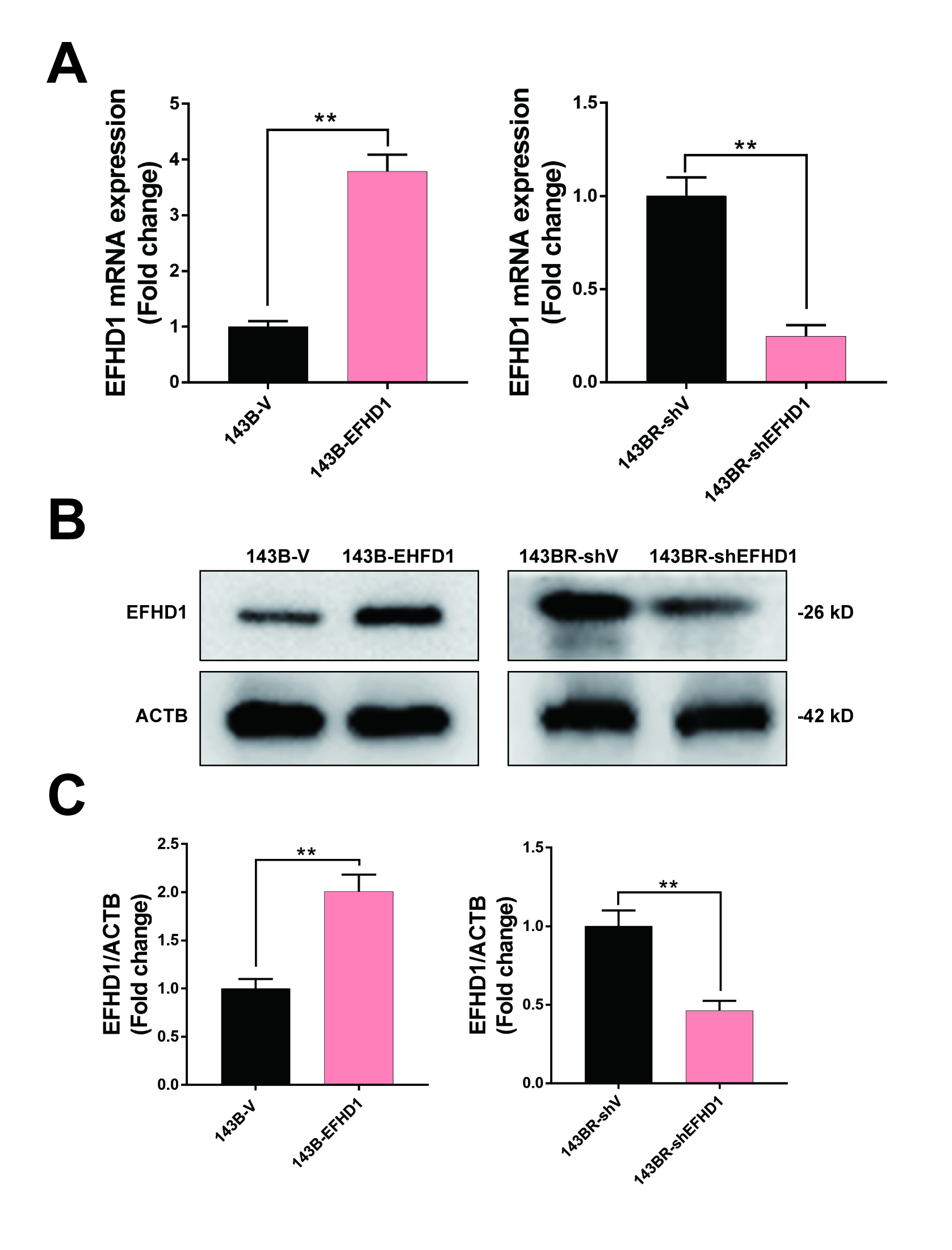

Supplement: Supplementary file 7 — Fig S4 qRT‒PCR and WB analysis of EFHD1 expression in overexpression and knockdown cells. A qRT‒PCR analysis of EFHD1 mRNA expression in 143B-EFHD1 and 143BR-shEFHD1 cells, N=3. B&C WB analysis of EFHD1 protein expression in 143B-EFHD1 and 143BR-shEFHD1 cells, N=3. The data are presented as the means ± SEMs; *P <0.05, **P <0.01. Supplementary file7 (TIF 2411 KB) [file 18_2024_5254_MOESM7_ESM.tif]

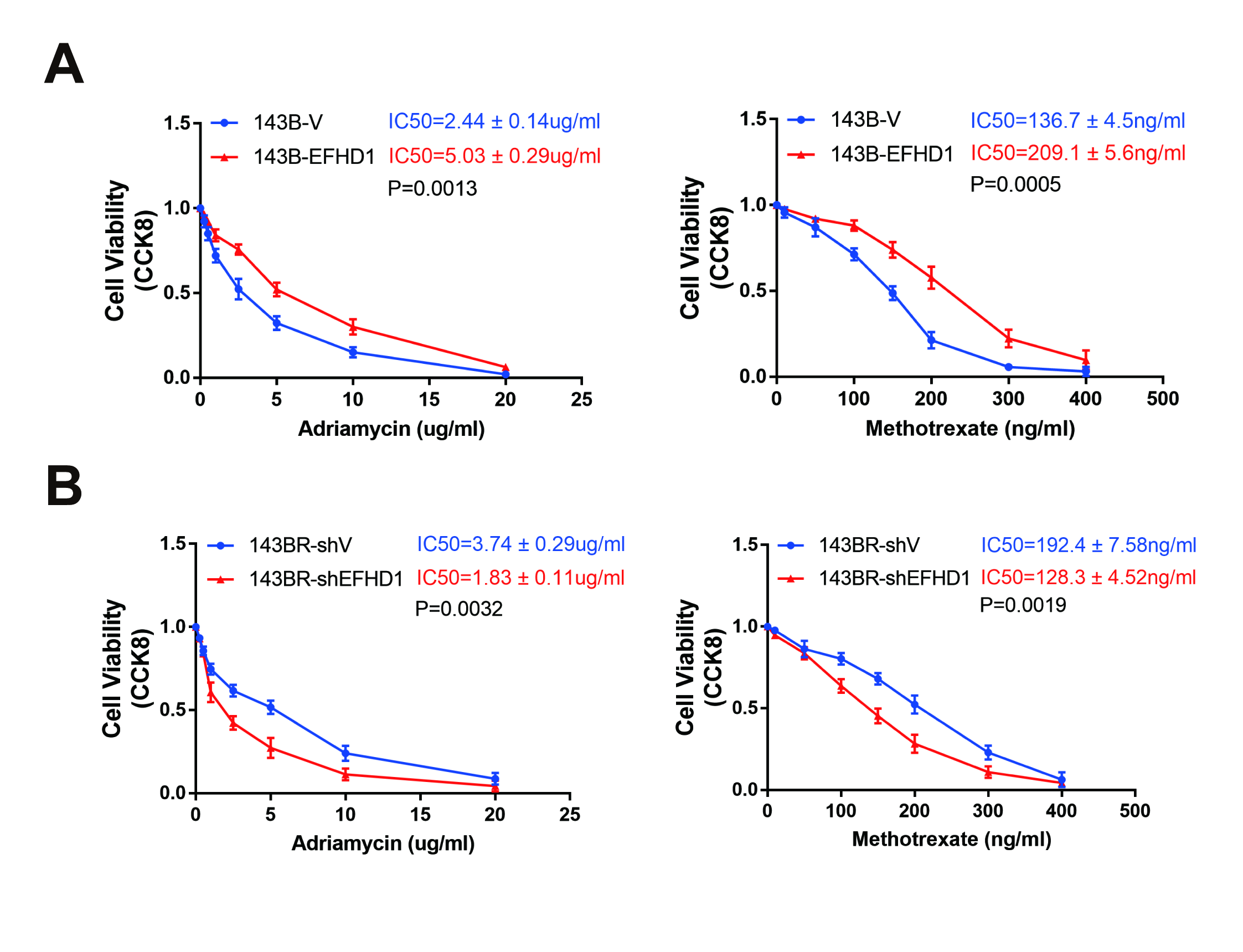

Supplement: Supplementary file 8 — Fig S5 EFHD1 promotes osteosarcoma cell resistance to adriamycin and methotrexate treatment. A Viability of 143B-V and 143B-EFHD1 cells after 24 h of adriamycin treatment (left) and methotrexate treatment (right), N=3. B Viability of 143BR-shV and 143BR-shEFHD1 cells after 24 h of adriamycin treatment (left) and methotrexate treatment (right), N=3. The data are presented as the means ± SEMs; *P <0.05, **P <0.01. Supplementary file8 (TIF 2091 KB) [file 18_2024_5254_MOESM8_ESM.tif]

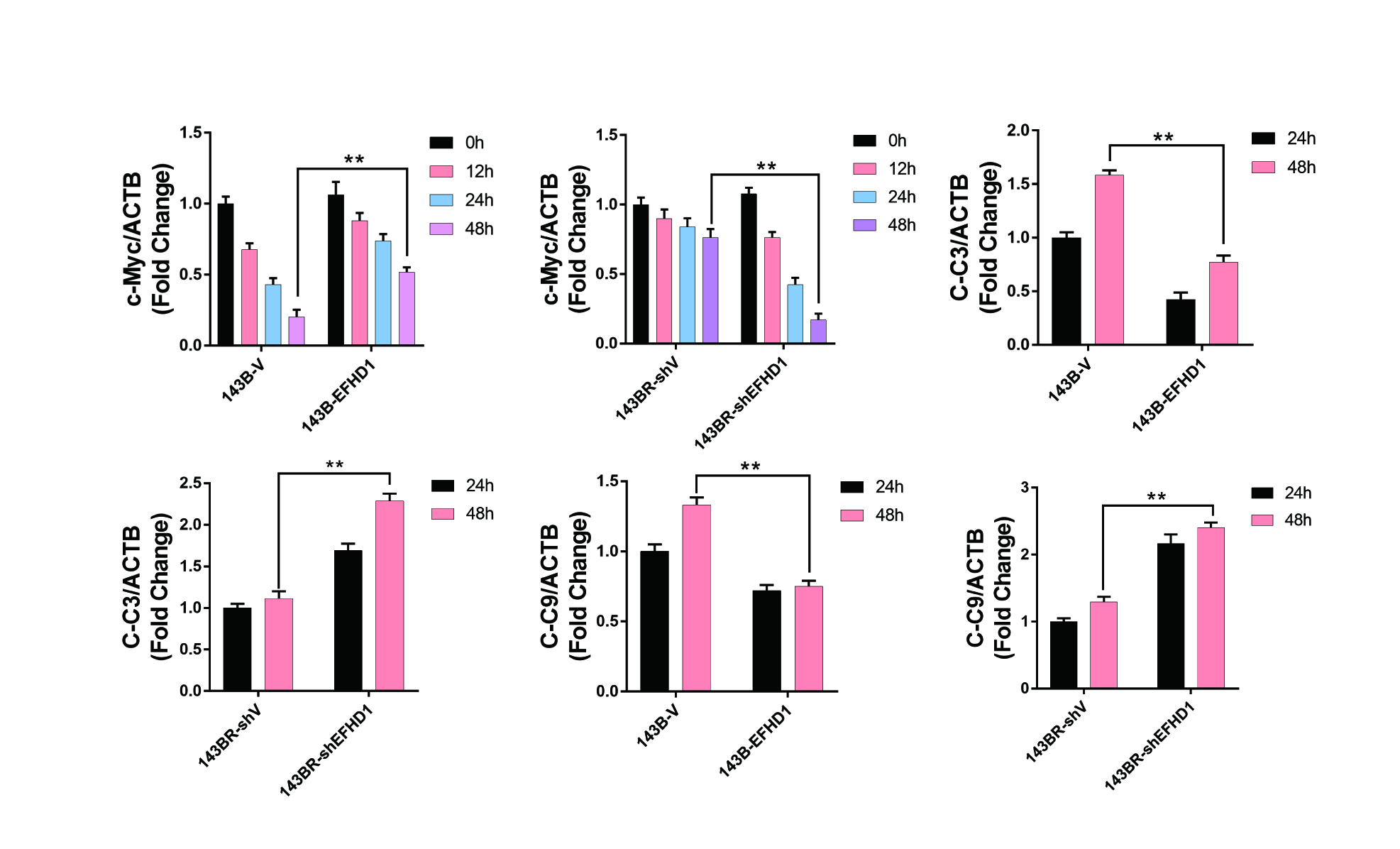

Supplement: Supplementary file 9 — Fig S6 Quantification of c-Myc, cleaved caspase 9 (C-C9), and cleaved caspase 3 (C-C3) expression based on WB. The data are presented as the means ± SEMs; *P <0.05, **P <0.01. Supplementary file9 (TIF 1603 KB) [file 18_2024_5254_MOESM9_ESM.tif]

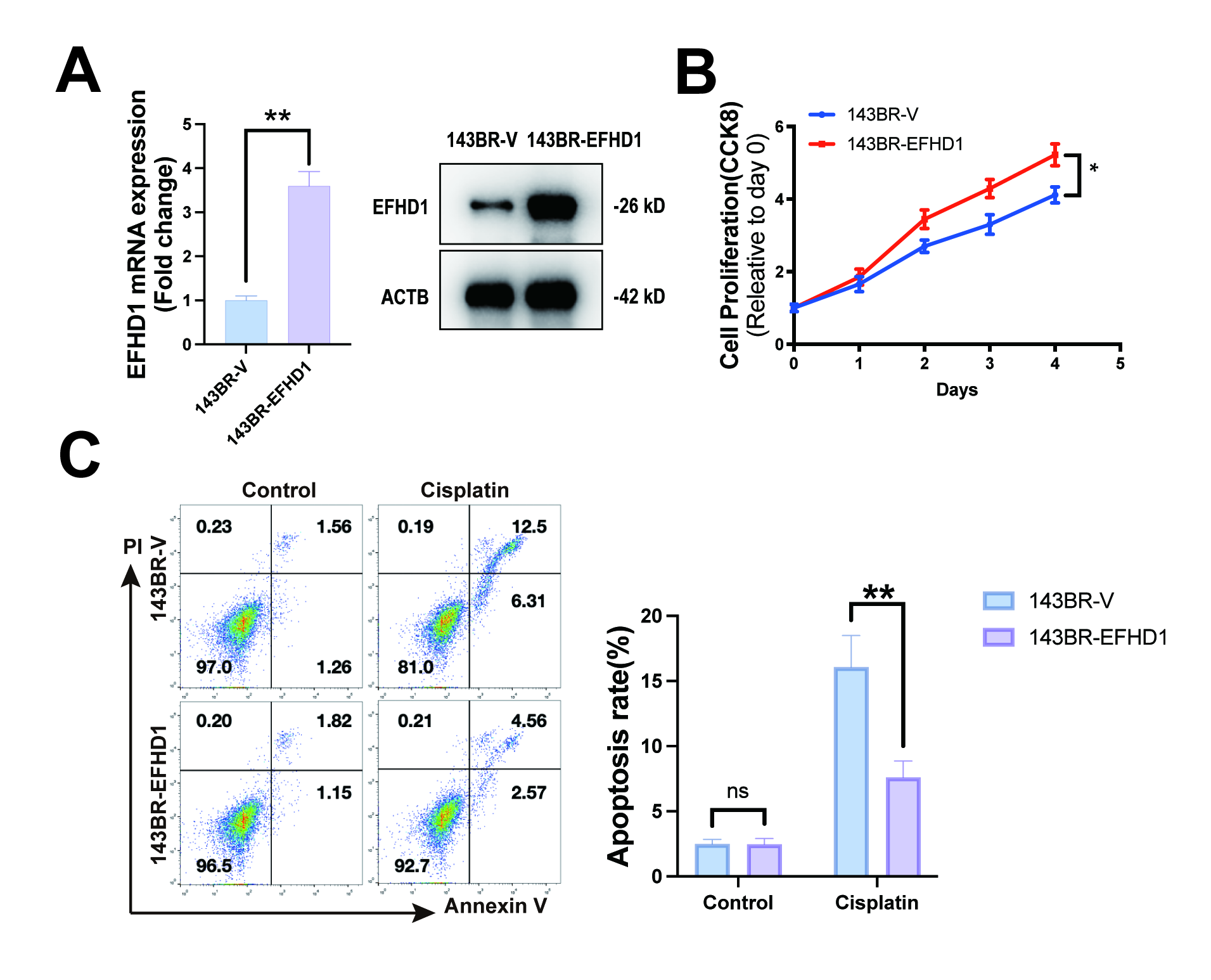

Supplement: Supplementary file 10 — Fig S7 EFHD1 promotes 143BR proliferation and chemoresistance in vitro. A qRT‒PCR and WB analysis of EFHD1 expression in 143BR-V and 143BR-EFHD1 cells, N=3. B The proliferation of 143BR-V and 143BR-EFHD1 cells was detected by CCK8 proliferation assay, N=3. C Apoptosis of 143BR-V and 143BR-EFHD1 cells was measured by flow cytometric analysis, N=3. The data are presented as the means ± SEMs; *P <0.05, **P <0.01. Supplementary file10 (TIF 2109 KB) [file 18_2024_5254_MOESM10_ESM.tif]

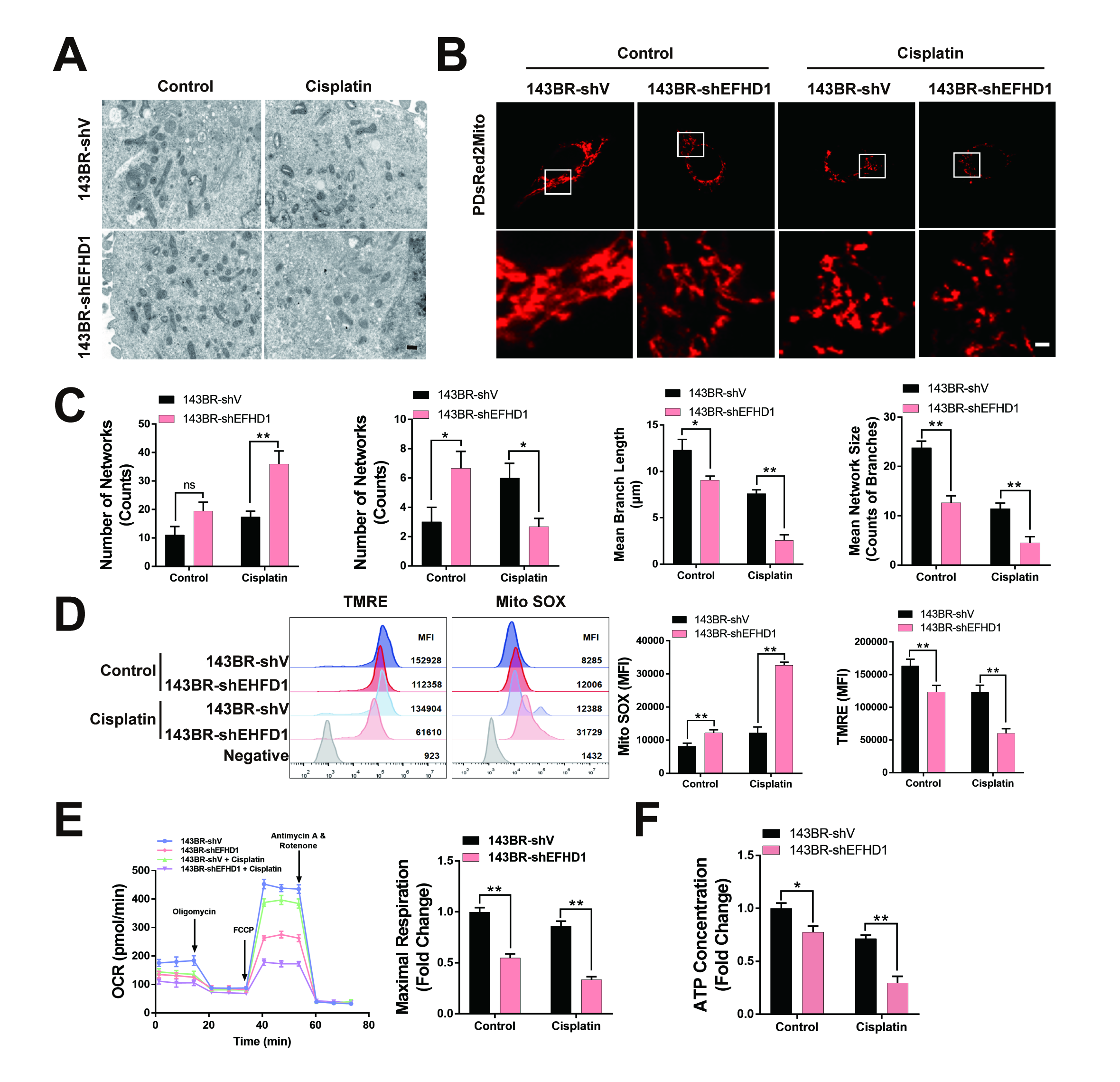

Supplement: Supplementary file 11 — Fig S8 EFHD1 knockdown in 143BR cells results in decreased mitochondrial function. A TEM images showing the mitochondrial morphology of 143BR-shV and 143BR-shEFHD1 cells treated with or without cisplatin. Scale bar=1 µm, N=3. B CLSM images showing the mitochondrial morphology of 143BR-shV and 143BR-shEFHD1 cells treated with or without cisplatin. Scale bar= 1 µm, N=3. C Histogram showing the results of mitochondrial network analysis of CLSM images. D Mitochondrial membrane potential (TMRE staining) and ROS production (MitoSOX staining) of 143BR-shV and 143BR-shEFHD1 cells treated with or without cisplatin were analyzed by flow cytometry, N=3. E Left: The OCR of 143BR-shV and 143BR-shEFHD1 cells was measured under basal conditions and in response to oligomycin, the mitochondrial decoupler FCCP and rotenone + antimycin after treatment with or without cisplatin. Right: the maximum OCR values were those achieved after FCCP uncoupling (maximum respiration), N=3. F ATP concentrations of 143BR-shV and 143BR-shEFHD1 cells were measured by an ATP lite 1step assay under control and cisplatin treatment conditions, N=3. The data are presented as the means ± SEMs; *P <0.05, **P <0.01. Supplementary file11 (TIF 6116 KB) [file 18_2024_5254_MOESM11_ESM.tif]

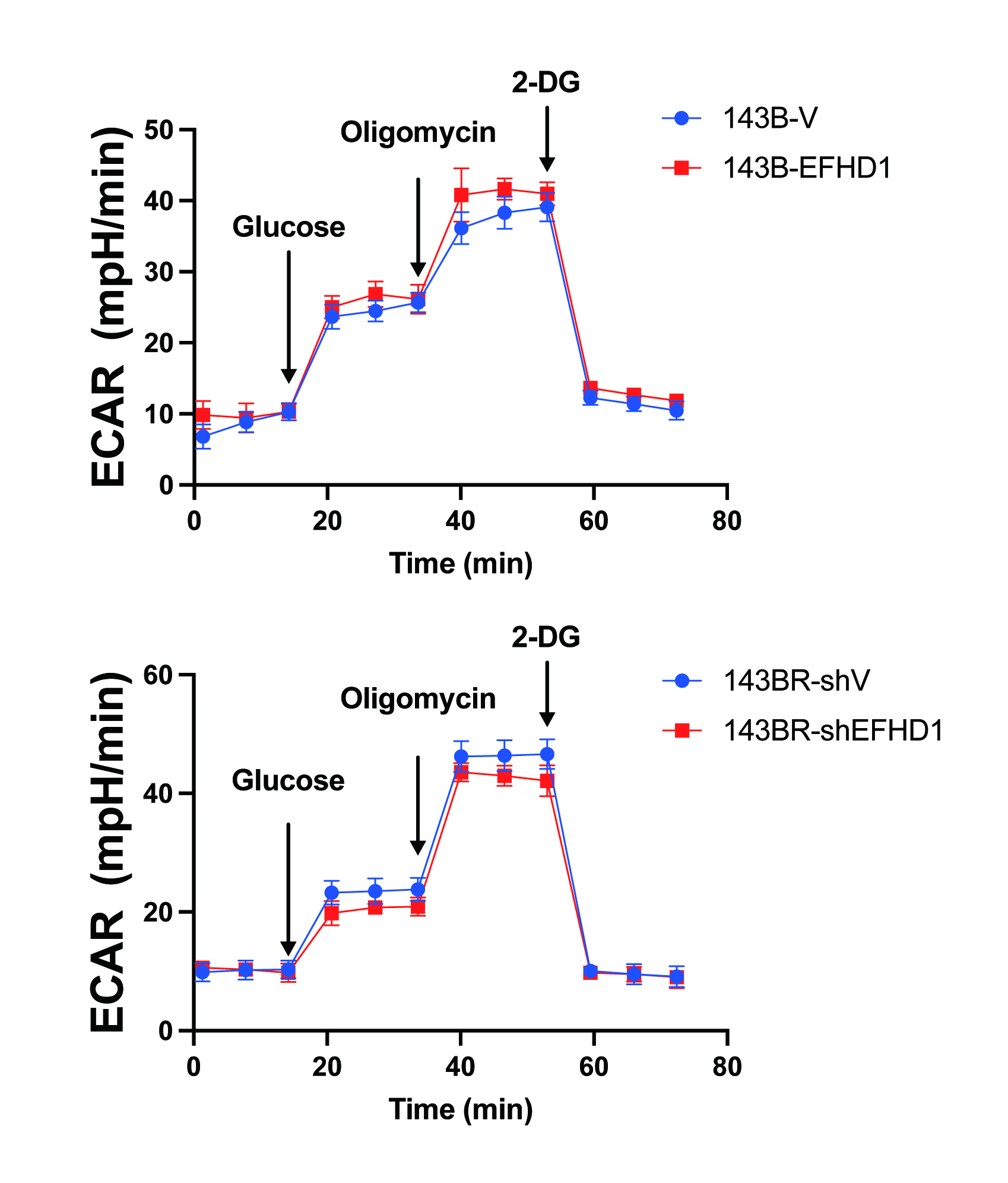

Supplement: Supplementary file 12 — Fig S9 The ECAR of 143B-V, 143B-EFHD1, 143BR-shV and 143BR-shEFHD1 cells was detected using an XF96 Extracellular Flux Analyzer. The data are presented as the means ± SEMs; *P <0.05, **P <0.01, N=3. Supplementary file12 (TIF 1417 KB) [file 18_2024_5254_MOESM12_ESM.tif]

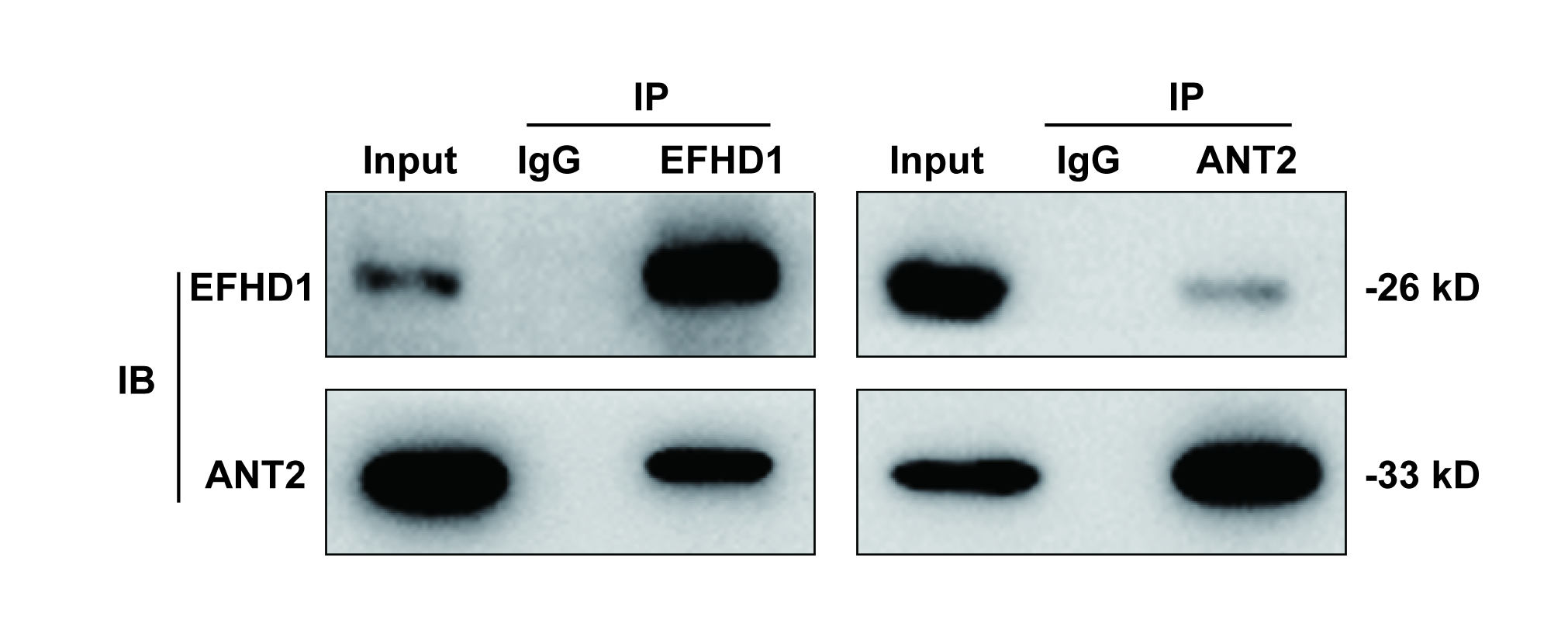

Supplement: Supplementary file 13 — Fig S10 IP/IB analysis was used to detect the interaction of EFHD1 with endogenous ANT2 in 143B cells. The data are presented as the means ± SEMs; *P <0.05, **P <0.01, N=3. Supplementary file13 (TIF 2175 KB) [file 18_2024_5254_MOESM13_ESM.tif]

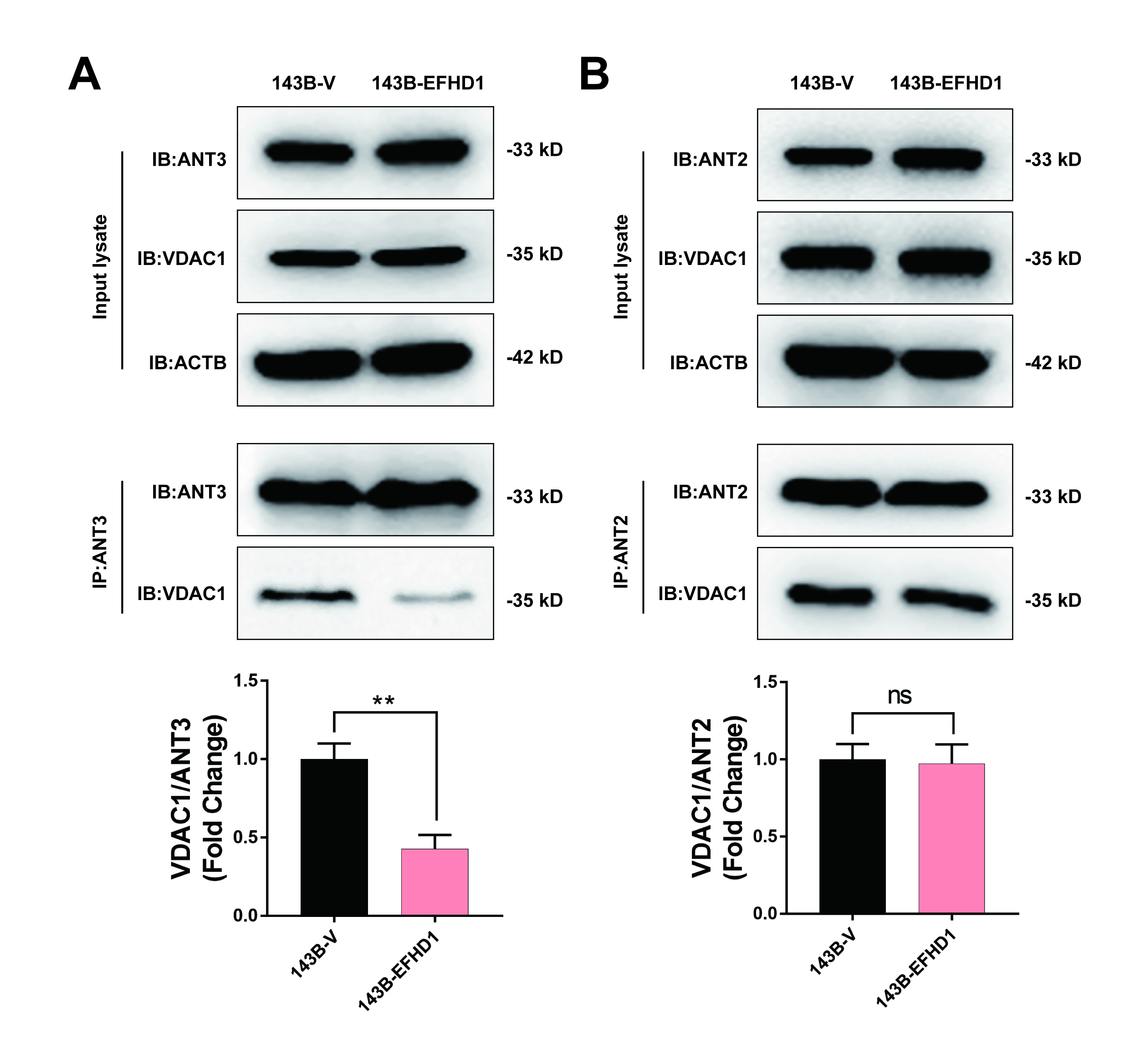

Supplement: Supplementary file 14 — Fig S11 Overexpression of EFHD1 in 143B cells inhibits ANT3-VDAC1 complex formation. A IP/IB was used to detect the formation of the ANT3-VDAC1 complex in 143B-V and 143B-EFHD1 cells, N=3. B IP/IB was used to detect the formation of the ANT2-VDAC1 complex in 143B-V and 143B-EFHD1 cells, N=3. The data are presented as the means ± SEMs; *P <0.05, **P <0.01. Supplementary file14 (TIF 3782 KB) [file 18_2024_5254_MOESM14_ESM.tif]

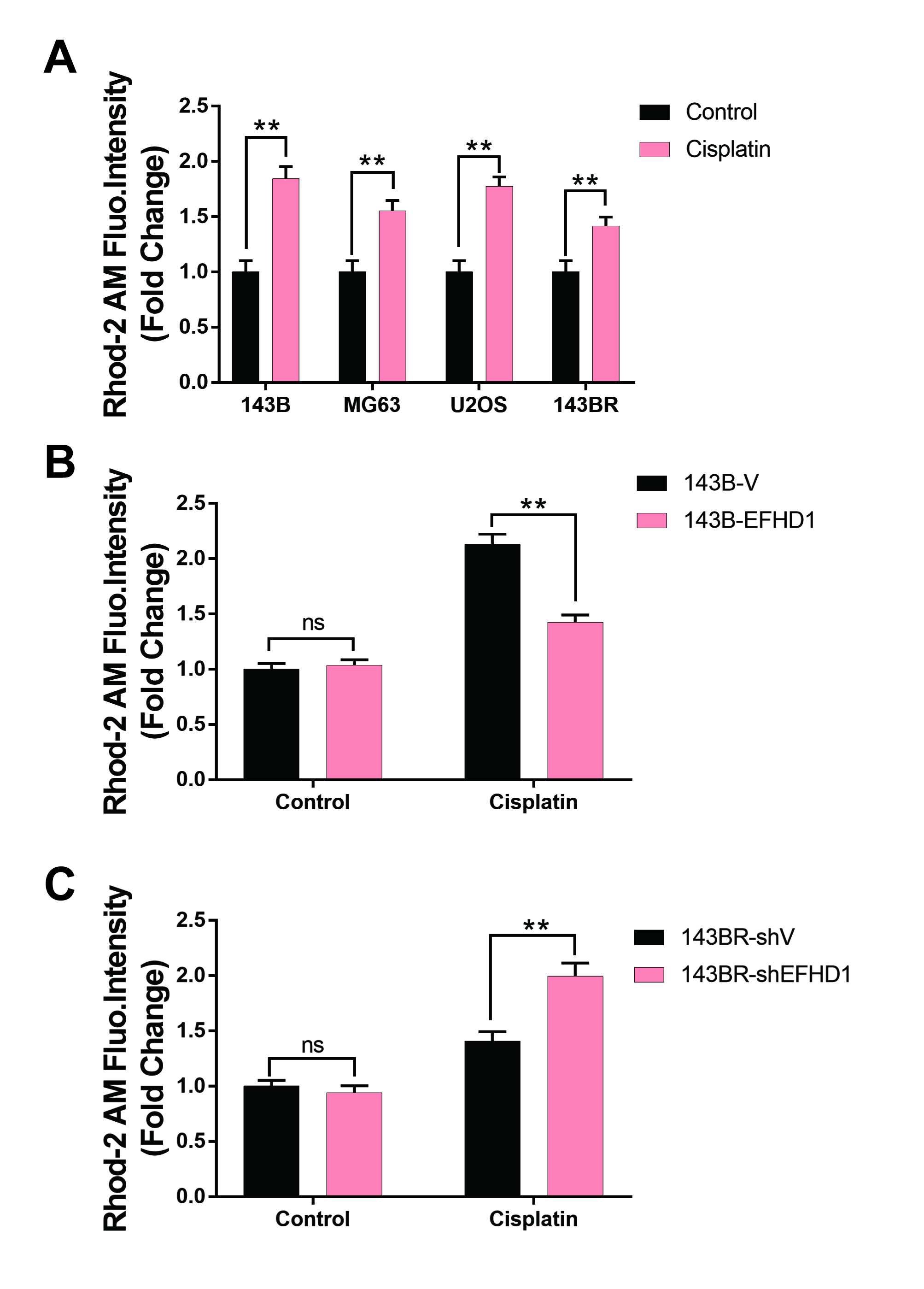

Supplement: Supplementary file 15 — Fig S12 EFHD1 reduces the cisplatin-induced accumulation of Ca2+ in the mitochondrial matrix. A The Rhod-2 AM probe was used to measure the mitochondrial Ca2+ concentrations in osteosarcoma cells after cisplatin treatment, N=3. B The Rhod-2 AM probe used to measure the mitochondrial Ca2+ concentrations in 143B-V and 143B-EFHD1 cells treated with/without cisplatin, N=3. C The Rhod-2 AM probe was used to measure the mitochondrial Ca2+ concentrations in 143BR-shV and 143BR-shEFHD1 cells treated with/without cisplatin, N=3. The data are presented as the means ± SEMs; *P <0.05, **P <0.01. Supplementary file15 (TIF 2174 KB) [file 18_2024_5254_MOESM15_ESM.tif]

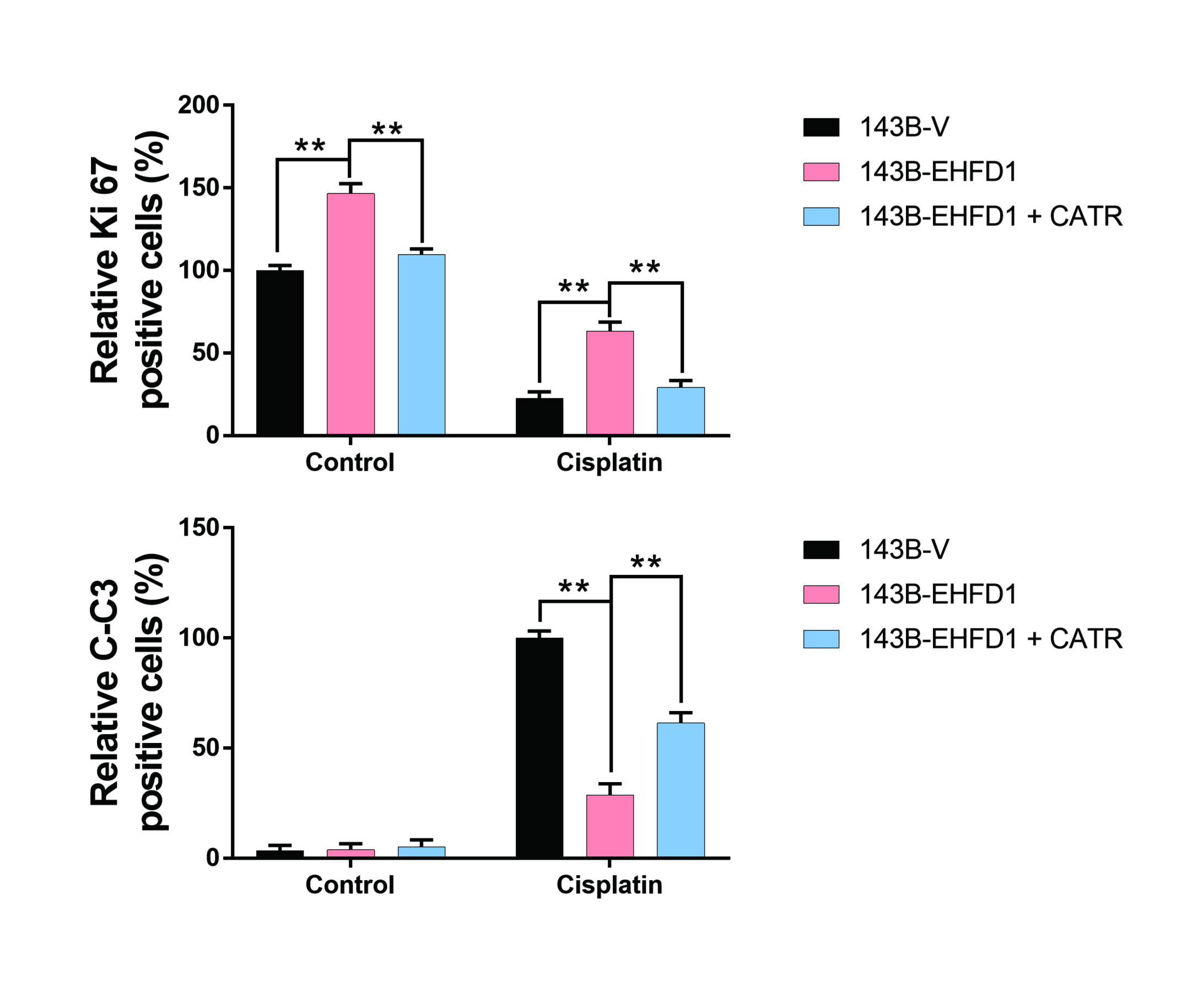

Supplement: Supplementary file 16 — Fig S13 Quantification of Ki-67 and C-C 3 expression based on IHC staining. The data are presented as the means ± SEMs; *P <0.05, **P <0.01, N=3. Supplementary file16 (TIF 1735 KB) [file 18_2024_5254_MOESM16_ESM.tif]

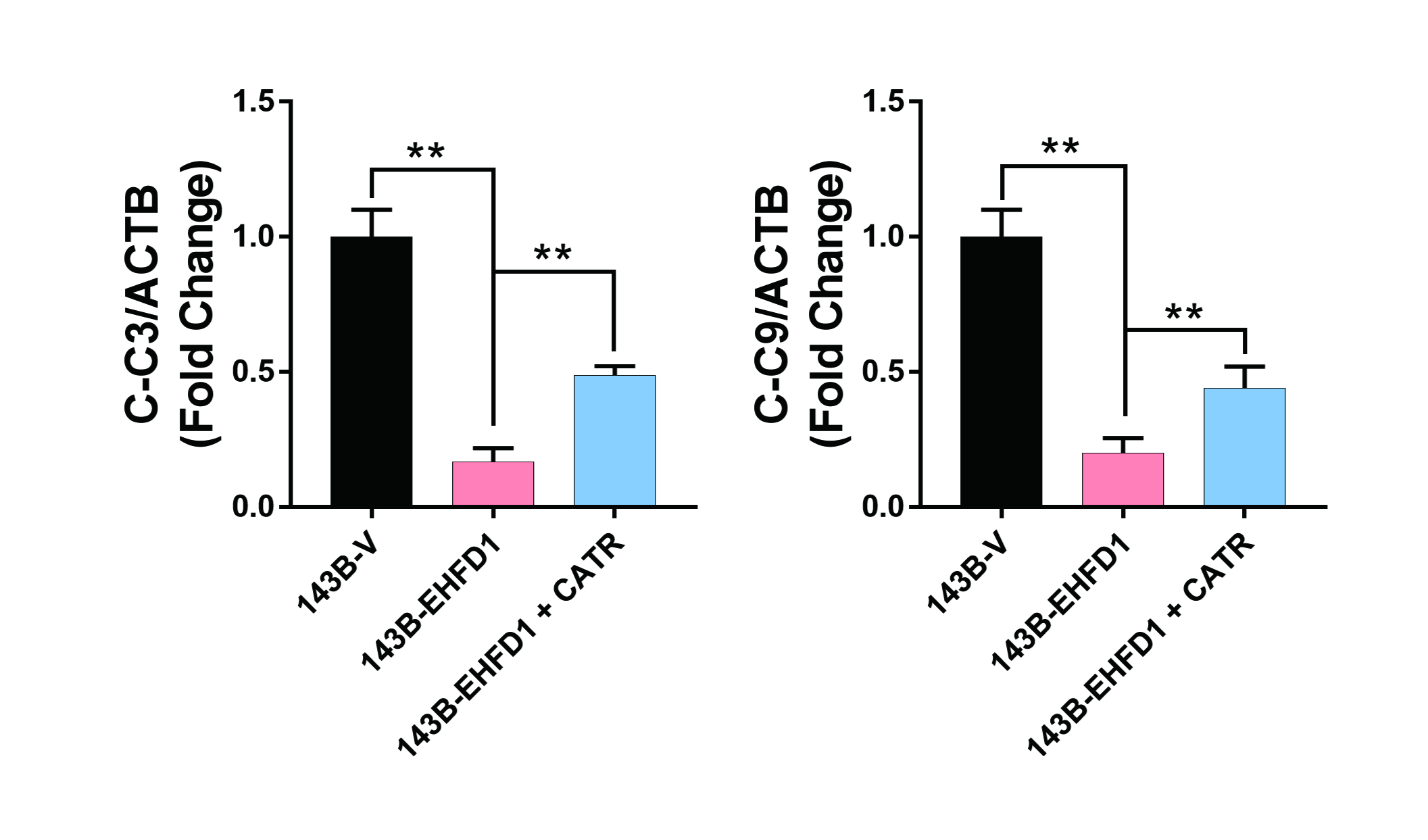

Supplement: Supplementary file 17 — Fig S14 Quantification of Ki-67 and C-C 3 protein expression based on WB. The data are presented as the means ± SEMs; *P <0.05, **P <0.01, N=3. Supplementary file17 (TIF 1294 KB) [file 18_2024_5254_MOESM17_ESM.tif]

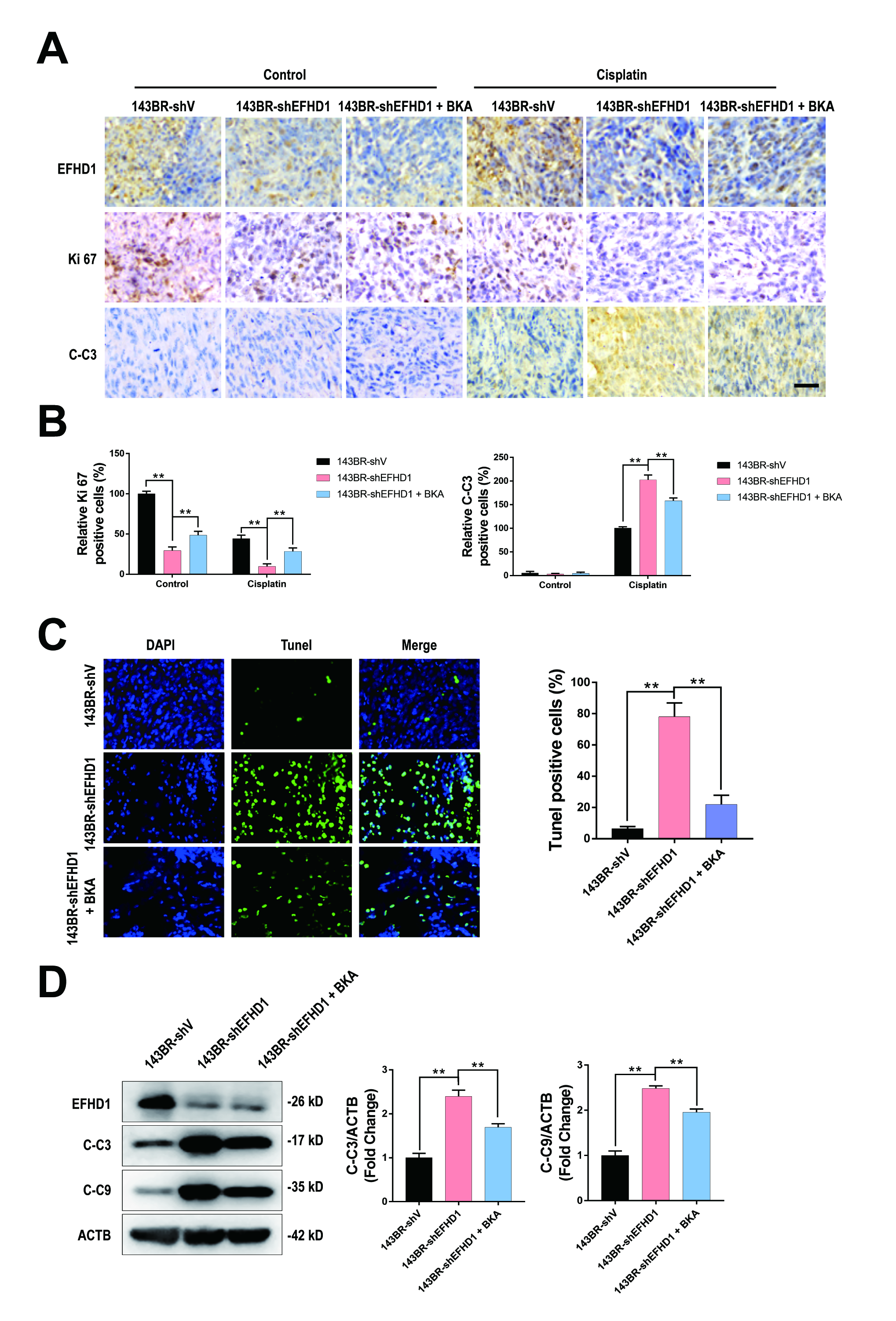

Supplement: Supplementary file 18 — Fig S15 BKA decreases the sensitivity of 143BR-shEFHD1 cells to cisplatin in vivo. A&B IHC staining analysis of Ki-67 and C-C 3 expression in 143BR xenograft tissues. Scale bar, 20 μm, N=5. C Apoptosis was analyzed by TUNEL assay (scale bar, 50 μm), N=5. D Apoptosis was analyzed by Western blotting, N=3. The data are presented as the means ± SEMs; *P <0.05, **P <0.01. Supplementary file18 (TIF 9234 KB) [file 18_2024_5254_MOESM18_ESM.tif]
